# Pectin under stress: Pectin methylesterases at the core of cell wall remodeling, integrity sensing, and signaling in plants

**DOI:** 10.1016/j.xplc.2026.101930

**Published:** 2026-05-25

**Authors:** Gabriele Pecatelli, Giulia Caminada, Daniele Coculo, Vincenzo Lionetti

**Affiliations:** 1 Dipartimento di Biologia e Biotecnologie “C. Darwin”, Sapienza Università di Roma, 00185 Rome, Italy

**Keywords:** plant stress, pectin methylesterase, pectin methylesterase inhibitors, pectin remodeling, damage-associated molecular patterns, cell wall integrity

## Abstract

Plant cell walls are dynamic signaling platforms that integrate mechanical, biochemical, and immune cues during development and environmental stress responses. Among wall polysaccharides, pectin has emerged as a central regulator of cell wall integrity (CWI) and stress-responsive signaling. Here, we highlight pectin methylesterases (PMEs) and their regulators as a coordinated modulatory system that enables spatiotemporal control of pectin remodeling during biotic and abiotic stress responses. We discuss recent discoveries regarding isoform-specific PME functions, proteolytic activation, and trafficking routes. During biotic interactions, PME activity promotes pectin remodeling for cell wall reinforcement. It also contributes to CWI sensing by supporting the generation of methanol and, indirectly, oligogalacturonides (OGs), and by enabling the formation of homogalacturonan (HG) structures that facilitate mechanosensor and receptor binding. Under abiotic stress, PME-driven de-methylesterification modulates cell wall stiffness, mechanics, porosity, and ion homeostasis. This review highlights PMEs as central regulators of pectin dynamics that underlie cell wall remodeling, signaling, and stress responses, identifies key knowledge gaps, and outlines future directions for enhancing plant stress resilience.

## Teaser

Pectin methylesterases (PMEs) have emerged as multifunctional regulators linking cell wall remodeling, mechanoperception, and stress signaling in plants. Through the coordinated actions of PME inhibitors, subtilases, apoplastic pH, and Ca^2+^ availability, PME activity dynamically controls pectin de-methylesterification, receptor complex organization, danger signal generation, wall mechanics, and ion buffering during biotic and abiotic stress responses.

## Introduction

As sessile organisms, plants are continuously exposed to biotic and abiotic stresses and rely on coordinated extracellular and intracellular responses to maintain fitness (Kong and Yang, 2023; Traubenik et al., 2024). The cell wall (CW) therefore acts as a dynamic, responsive interface that integrates environmental signals and helps orchestrate defense responses (Mohnen, 2008; Pogorelko et al., 2013; Vicré and Lionetti, 2023; Molina et al., 2024). The CW is a dynamic structure composed primarily of polysaccharides, including cellulose, hemicellulose, and pectin, together with phenolic compounds, proteins, ions, and water (Delmer et al., 2024). This structural complexity underpins the concept of CW integrity (CWI), defined as the functional state of the wall that ensures proper cellular homeostasis and growth. CWI is preserved through surveillance systems that sense structural perturbations and activate downstream signaling pathways, thereby triggering compensatory responses to restore wall functionality during development and under stress conditions (Engelsdorf et al., 2018; Bacete and Hamann, 2020; Baez et al., 2022).

Among CW components, pectin plays a central role in CWI surveillance and maintenance (Lionetti et al., 2017; Bhandari et al., 2025). This galacturonic acid-rich polysaccharide family comprises several structural domains, including homogalacturonan (HG), rhamnogalacturonans I and II, and xylogalacturonan (Lionetti et al., 2007; Delmer et al., 2024). HG is a linear homopolymer of α-1,4-linked D-galacturonic acid (GalA) residues. HG is synthesized in the Golgi apparatus and contributes to CWI maintenance and immunity (Bethke et al., 2016; Du et al., 2022). The HG backbone is synthesized by galacturonosyltransferases (GAUTs), which transfer GalA from UDP-GalA to the growing polymer (Atmodjo et al., 2011; Engle et al., 2022). HG is methylesterified at the C-6 position by HG methyltransferases (HG-MTs), which use S-adenosylmethionine (SAM) as the methyl donor, with SAM being imported into the Golgi lumen by Golgi SAM transporters (GoSAMTs) (Ibar and Orellana, 2007; Mouille et al., 2007; Temple et al., 2022) (Figure 2).

**Figure 1:**
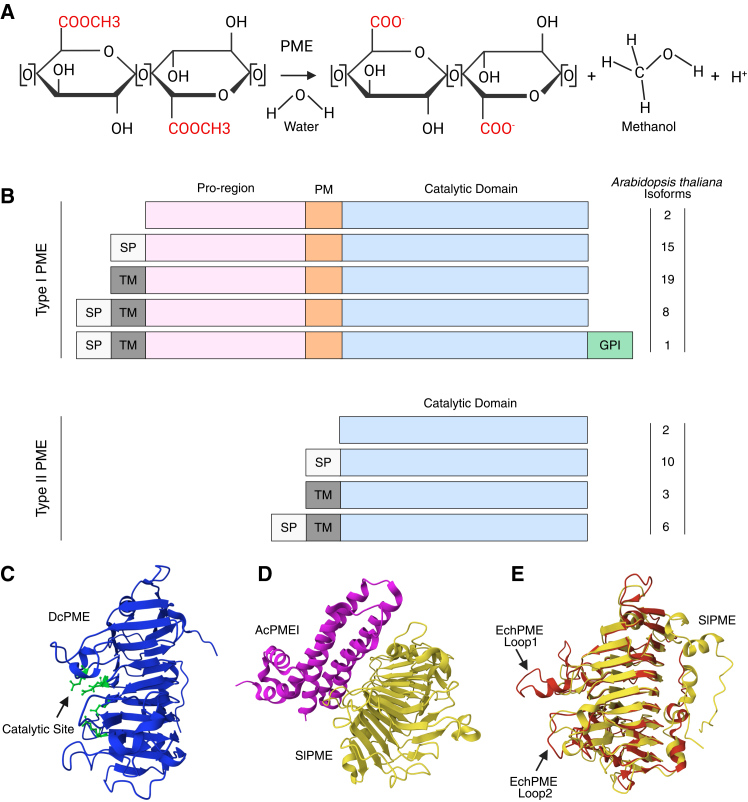
The structure and activity of plant PMEs. **(A)** Schematic representation of the reaction catalyzed by PMEs showing the hydrolysis of methyl ester groups in HG, which results in the formation of free carboxyl groups and the release of methanol and H^+^. **(B)** Domain organization of plant PME isoforms. Type I PMEs contain an N-terminal pro-domain followed by the catalytic domain, whereas type II PMEs consist solely of the catalytic domain. SPs, TM domains, and GPI anchors are indicated; numbers flanking each class indicate the number of isoforms identified in *Arabidopsis thaliana*. **(C)** Three-dimensional structure of *Daucus carota PME* (DcPME), displaying the characteristic right-handed parallel β-helix fold and highlighting the substrate-binding cleft that accommodates HG. **(D)** Structure of the PME-PMEI complex: *Actinidia chinensis* AcPMEI (magenta) in complex with *Solanum lycopersicum* SlPME (yellow). **(E)** Structural comparison of *Solanum lycopersicum* SlPME (yellow) and *Erwinia chrysanthemi* EchPME (orange), showing differences in surface loops indicated by black arrows. PME: pectin methylesterase; HG: homogalacturonan; SP: signal peptide; TM: transmembrane domain; GPI: glycosylphosphatidylinositol.

These activities establish the initial degree and pattern of HG methylesterification prior to secretion, thereby providing the biochemical framework for subsequent HG remodeling in the apoplast. CWI is maintained through the integration of biosynthesis and continuous post-synthetic modification of wall polymers, particularly under stress conditions (Rui and Dinneny, 2020; Swaminathan et al., 2021, 2022). Post-synthetic remodeling is regulated by multiple factors, including pectin-modifying enzymes, particularly pectin methylesterases (PMEs) (Lionetti et al., 2010; Yang et al., 2018). PMEs catalyze the hydrolysis of methyl ester groups at the C-6 carboxyl position of GalA residues in HG, releasing methanol and generating free carboxyl groups along the pectin backbone (Figure 1A). This activity affects both the degree and spatial pattern of HG methylesterification (Lionetti et al., 2012; Wu et al., 2018). PMEs belong to the carbohydrate esterase family 8 (CE8; CAZy database) and are encoded by multigene families. They are involved in a wide range of developmental processes, including organ growth, stomatal function, and reproduction, often acting in coordination with hormonal signaling pathways (Ren and Kermode, 2000; Bosch and Hepler, 2005; Peaucelle et al., 2011; Amsbury et al., 2016). This developmental context should be considered to better understand PME functions during stress responses and to interpret their underlying mechanisms.

PMEs play a critical role in multiple plant–pathogen interactions and responses to abiotic stress (Lionetti et al., 2012; Wu et al., 2018). PME-mediated pectin remodeling can promote CW reinforcement or loosening in an isoform- and context-dependent manner (Willats et al., 2001; Del Corpo et al., 2020). PMEs can contribute to CWI sensing and surveillance both by generating HG domains that support sensor binding and by promoting the release of danger signals, including oligogalacturonides (OGs) and methanol (MeOH) (Osorio et al., 2008; Komarova et al., 2014; Liu et al., 2024). Under abiotic stress, PME-mediated modulation of pectin charge and Ca^2+^-binding capacity influences mechanical wall properties and ion homeostasis (Wu et al., 2018; Coculo and Lionetti, 2022; Jiang et al., 2025).

These multifaceted functions require tight spatial and temporal control of PME trafficking, post-secretory maturation, activity, and function, achieved through coordinated regulation by PME inhibitors (PMEIs) (Coculo and Lionetti, 2022), subtilisin-like proteases (subtilases; SBTs) (Senechal et al., 2014; Coculo et al., 2023), and apoplastic pH (Hocq et al., 2024). In this review, we highlight PMEs as central modulators of pectin dynamics linking CW remodeling, signaling, and plant responses to biotic and abiotic stresses.

## Structural basis of PME targeting, trafficking, and function

**Figure 2:**
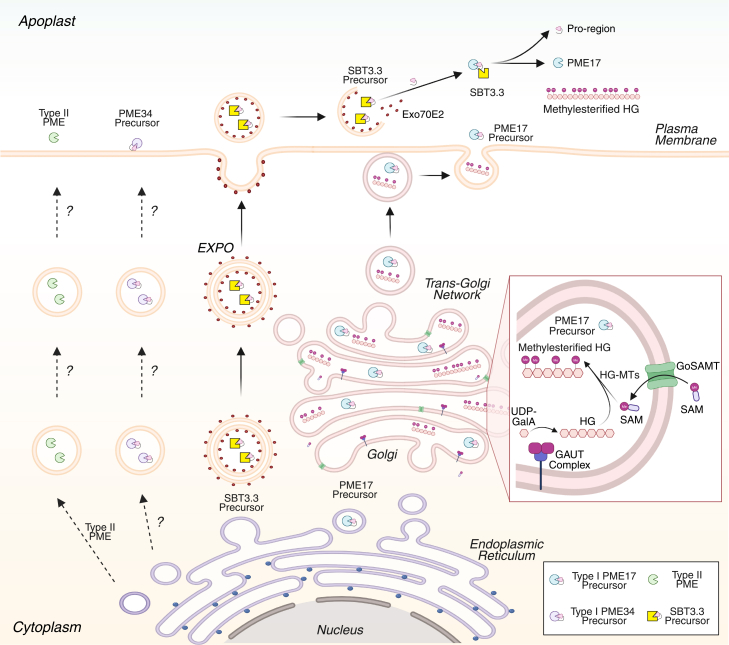
Intracellular trafficking, processing, and activation of PMEs. Signal peptides and TM domains contribute to the intracellular targeting and secretion of PMEs. The PME17 precursor, which contains a signal peptide but lacks a TM domain, follows the conventional endoplasmic reticulum-Golgi pathway and is secreted into the apoplast as an inactive precursor, where it is activated by the subtilase SBT3.3. In contrast, the PME34 precursor lacks a signal peptide but contains a TM domain, resulting in transient plasma membrane localization prior to apoplastic release. The SBT3.3 precursor is secreted via an unconventional pathway involving EXPOs, which may enable temporal control of PME activation. The secretion pathway of type II PMEs remains to be characterized. The right panel shows HG biosynthesis in the Golgi by GAUTs using UDP-GalA, followed by methyl modifications catalyzed by HG-MTs. Methyl esterification requires SAM as the methyl donor, which is imported into the Golgi by GoSAMTs, generating HG polymers with variable degrees of methylesterification. Dashed arrows and question marks indicate putative or unresolved trafficking routes. PME: pectin methylesterase; TM: transmembrane domain; EXPOs: exocyst-positive organelles; HG: homogalacturonan; GAUTs: galacturonosyltransferases; GalA: D-galacturonic acid; HG-MTs: HG methyltransferases; SAM: S-adenosylmethionine; GoSAMTs: Golgi SAM transporters.

PME structural features, together with post-translational modifications, are key determinants of intracellular trafficking, localization, and deployment under stress conditions (Mareck et al., 2012; Canut et al., 2016). Plant PMEs are classified into two main types based on their domain organization (Coculo and Lionetti, 2022) (Figure 1B). Type I PMEs (Group 2; 45 isoforms in *Arabidopsis thaliana*) include enzymes that are synthesized as inactive precursors with an N-terminal pro-domain. This pro-region, which is structurally related to PMEIs, acts as an intramolecular inhibitor that prevents premature PME activity (Del Corpo et al., 2020). Although some PMEs can be secreted as precursors, the presence and proper processing of the pro-region in specific type I PMEs contribute to correct targeting and secretion to the CW (Bosch and Hepler, 2005; Wolf et al., 2009; Li et al., 2022). Type II PMEs (Group 1; 21 isoforms in *A. thaliana*) comprise proteins containing only the catalytic domain. In contrast to type I PMEs, much less is known about the biogenesis and trafficking of type II isoforms (Figure 2).

Both PME types may contain an N-terminal signal peptide that directs entry into the secretory pathway and/or a transmembrane domain that mediates membrane association, although some lack one or both elements (Micheli, 2001; Bosch and Hepler, 2005) (Figure 1B). Representative PME isoforms highlight the diversity of trafficking strategies. AtPME17, a type I PME and a central regulator of CW-based immunity and marker of pathogenesis, contains a signal peptide but lacks a transmembrane domain (Lionetti et al., 2012; Del Corpo et al., 2020; Coculo et al., 2023). It localizes to the endoplasmic reticulum and Golgi before secretion into the apoplast, where it likely acts as a soluble and mobile enzyme that enables rapid CW remodeling during defense activation (Figure 2). By contrast, *AtPME34*, a type I PME highly expressed in guard cells and required for heat-stress tolerance, contains a transmembrane domain but lacks a signal peptide. It localizes to the apoplast-facing side of the plasma membrane before release, suggesting that membrane anchoring spatially organizes PME activity (Huang et al., 2017) (Figure 2). By contrast, AtPME31, a type I PME predicted to lack both signal peptide and transmembrane domains, localizes to the cytosol and peroxisomes and does not traffic to the CW. Collectively, these findings indicate that the signal peptide is a determinant of CW targeting, whereas transmembrane domains primarily confer membrane retention and spatial control of PME activity. Additional structural determinants can modulate PME trafficking. In *Nicotiana tabacum*, N-glycosylation of NtPPME1 at N482 is required for transport from Golgi-derived vesicles to the apoplast, bypassing the trans-Golgi network (Weng et al., 2025). This unconventional secretory pathway highlights an additional layer of spatial control over pectin remodeling. In addition, *AtPME35*, a type I PME, is predicted to carry a glycosylphosphatidylinositol anchor, which could enable stable attachment to the plasma membrane (Xu et al., 2022; Zhou, 2022). Overall, distinct combinations of structural features and post-translational modifications across PME isoforms direct their trafficking and targeting, thereby controlling the spatial degree and pattern of pectin methylesterification as part of an effective strategy for stress responses.

The resolution of three-dimensional PME structures has revealed key features that influence PME substrate specificity and mode of action. Tertiary structures of *Daucus carota* and *Solanum lycopersicum* PMEs have been resolved by X-ray crystallography, providing insights into catalytic mechanisms and functional specialization (Johansson et al., 2002; Di Matteo et al., 2005; Ciardiello et al., 2008). Plant PMEs share a conserved right-handed parallel β-helix fold with a surface cleft that accommodates HG and a highly conserved catalytic site (e.g., Q113, Q135, D136, D157, and R225 in DcPME numbering), where D157 acts as a nucleophile, and D136 participates in proton transfer and MeOH release (Figure 1C). Structural models of PME-pectin interaction suggest that plant PMEs operate in a processive mode of action (Kim et al., 2018). Variations in cleft geometry and electrostatic properties, together with apoplastic pH, likely contribute to distinct modes of action of PMEs (Mercadante et al., 2014; Sénéchal et al., 2017; Hocq et al., 2024). Structures of PMEIs and PME-PMEI complexes provide insights into the molecular basis of catalysis and inhibition. AtPMEI1 adopts a four-helix bundle structure stabilized by conserved disulfide bridges and displays a dimeric arrangement (Hothorn et al., 2004a, 2004b). Complex structures reveal a 1:1 SlPME-AcPMEI interaction in which the inhibitor occludes the catalytic cleft, blocking substrate access (Di Matteo et al., 2005; Ciardiello et al., 2008) (Figure 1D). These findings support a PME-centered model of inhibition, in which enzyme surface geometry determines PMEI binding. Interestingly, microbial PMEs are not inhibited by plant PMEIs. While plant PMEs are structurally conserved, bacterial PMEs display variations in substrate-binding clefts, including extended loops and altered geometry, likely affecting substrate accessibility, mode of action, and PMEI interaction (Markovic and Janecek, 2004) (Figure 1E). These differences provide a framework for engineering PME and PMEI structural features to enhance plant resistance under stress conditions. Consistent with this idea, recent studies have shown that AlphaFold-guided redesign of PMEIs can expand their inhibitory spectrum toward pathogen-derived PMEs (Xia et al., 2024).

## Functional plasticity of PMEs in biotic stress: From cell wall reinforcement to immune signaling

**Figure 3:**
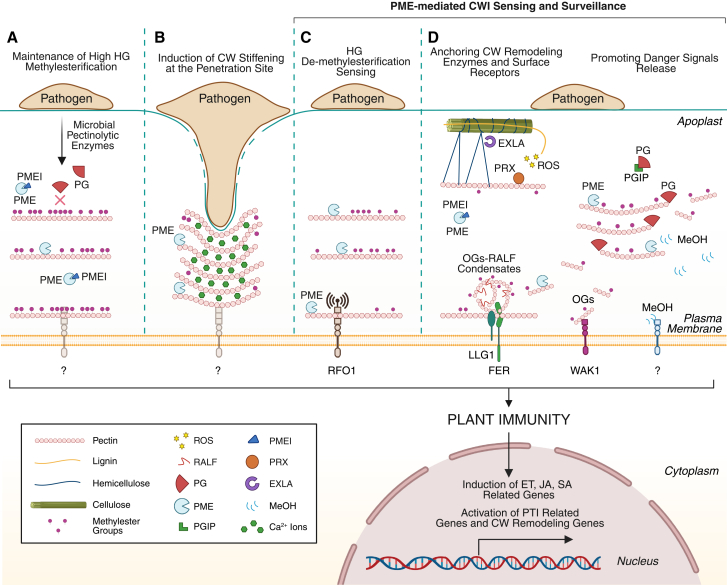
Mechanisms of PME-mediated defense responses during biotic stress. **(A)** Protection of HG by maintaining high levels of methylesterification, ensured by the coordinated activities of PMEs and PMEIs, thereby limiting the accessibility of microbial pectinolytic enzymes, such as PGs. **(B)** Localized CW reinforcement through HG de-methylesterification and Ca^2+^-mediated cross-linking between adjacent HG chains. **(C)** HG de-methylesterification is perceived by specific sensors. **(D)** On the left, de-methylesterified HG acts as a scaffold for the anchoring of CW-associated enzymes and surface receptors, thereby contributing to the spatial organization of CW remodeling and signaling. On the right, PME activity promotes the generation of signaling molecules, such as OGs and MeOH, and the activation of receptor-mediated signaling pathways. These perception events activate hormonal signaling pathways, which in turn coordinate the induction of PTI-related genes and CW remodeling genes. Question marks indicate putative or not yet fully resolved sensing mechanisms. PME: pectin methylesterase; PMEIs: PME inhibitors; HG: homogalacturonan; PG: polygalacturonases; PGIP: polygalacturonase-inhibiting proteins; OGs: oligogalacturonides; MeOH: methanol; RFO1: RESISTANCE TO FUSARIUM OXYSPORUM 1; PRXs: peroxidases; EXLAs: expansin-like A proteins; ROS: reactive oxygen species; RALF: RAPID ALKALINIZATION FACTOR; FER: FERONIA; LLG1: LORELEI-LIKE GPI-ANCHORED PROTEIN 1; WAK1: WALL-ASSOCIATED KINASE 1; ET: ethylene; JA: jasmonic acid; SA: salicylic acid; PTI: pattern-triggered immunity.

Plant pathogens adopt distinct lifestyles that shape their interaction with the host. Necrotrophs actively kill host cells to exploit dead tissue, whereas biotrophs depend on living cells and therefore evade or suppress host immune responses while maintaining host viability; hemibiotrophs combine these strategies by transitioning from an initial biotrophic phase to a necrotrophic phase (Kim et al., 2022). Despite these differences, successful colonization requires the secretion of a wide range of microbial CW-degrading and CW-modifying enzymes (Engelsdorf et al., 2018). The control of pectin integrity lies at the heart of a molecular tug-of-war between the maintenance of plant physiological integrity and the onset of disease (Bellincampi et al., 2014; Munzert and Engelsdorf, 2025). In this context, plant PMEs act as central mediators of CW remodeling and surveillance (Figure 3; Table 1).

**Table 1:** *Arabidopsis thaliana* PME isoforms mainly involved in biotic and abiotic stress responses.

Locus	PME isoform	PME type	Signal peptide	Transmembrane domain	Interacting organism(s) or abiotic stress	References
**Biotic stress**						
At1g11580	PMEPCRA (also known as PME18)	I	-	+	*P*. *brassicae*	(Stefanowicz et al., 2021)
At2g45220	PME17	I	+	+	*B*. *cinerea*	(Del Corpo et al., 2020)
At3g14310	PME3	I	-	+	*P*. *carotovorum*, *B*.*cinerea*, *H*. *schachtii*	(Hewezi et al., 2008; Raiola et al., 2011; Bohlmann and Sobczak, 2014)
At3g27980	PME30	I	+	-	*B. subtilis*	(Lakshmanan et al., 2013)
**Abiotic stress**				Type of abiotic stress		
At1g23200	PME6	I	-	-	drought	(Amsbury et al., 2016)
At2g26440	PME12	I	+	-	heat	(Wu et al., 2025)
At2g45220	PME17	I	+	+	Al	(Geng et al., 2017)
At3g14310	PME3	I	-	+	Zn	(Weber et al., 2013)
At3g29090	PME31	II	-	-	salt and drought	(Yan et al., 2018; Zhang et al., 2023)
At3g49220	PME34	I	-	+	heat	(Huang et al., 2017)
At4g02330	PME41	I	+	-	cold	(Qu et al., 2011)
At5g04960	PME46	I	-	+	Al	(Geng et al., 2017)
At5g04970	PME47	I	+	-	salt	(Yan et al., 2018; Zhou et al., 2024)
At5g19730	PME53	II	+	-	heat	(Wu et al., 2022)
At5g20860	PME54	I	+	-	Al	(Geng et al., 2017)

### Hormonal and transcriptional regulation of PMEs in pathogen responses

Activation of specific plant PME isoforms with distinct temporal patterns across multiple pathosystems highlights the importance of tightly regulated pectin de-methylesterification and associated signaling during pathogen attack (Lionetti et al., 2012; Bethke et al., 2014; Del Corpo et al., 2020) (Figure 3). Type I PMEs appear to be particularly important, and their expansion across plant lineages likely reflects evolutionary selection for tight regulatory control, allowing PME precursors to be activated in a timely manner during pathogen challenge (Coculo and Lionetti, 2022; Coculo et al., 2023). At*PMEPCRA*, *AtPME3*, *AtPME17*, *AtPME20*, *AtPME21*, and *AtPME41* are induced at distinct stages of infection, whereas *AtPME1* and *AtPME34* are repressed (Lionetti et al., 2017). These expression patterns suggest isoform-specific functions during pathogen colonization.

Stress-responsive PME genes are induced by multiple defense-associated phytohormones, including abscisic acid (ABA), jasmonic acid (JA), salicylic acid (SA), and ethylene (ET) (Nafisi et al., 2015; Del Corpo et al., 2020; Sun et al., 2022) (Figure 3). Functional analyses show that the loss-of-function *pme17* mutants exhibit enhanced susceptibility to *Botrytis cinerea* and reduced expression of the JA/ET-dependent defense gene *PLANT* DEFENSIN *1.2* (*PDF1.2*). In line with this, PME-mediated de-methylesterification releases MeOH, which stimulate ET biosynthesis and thereby reinforce JA/ET-dependent defense responses (Tran et al., 2018). Overall, PME activity acts as a mediator of JA- and ET-dependent pathways during plant-pathogen interactions (Bethke et al., 2014; Taurino et al., 2014).

In addition to hormonal regulation, transcriptional control of specific PME genes also contributes to the modulation of pectin remodeling during immune responses. In *Vitis vinifera*, *VvPME10*, an ortholog of *AtPME17*, primes early defense responses against *B. cinerea* and improves resistance to fungal infection (Lagrèze et al., 2025). *VvPME10* expression is activated by the defense-related transcription factor WRKY03, highlighting the importance of transcriptional control in PME-mediated immunity (Eulgem et al., 2000). However, this regulatory layer remains relatively underexplored. Insights from developmental studies may help bridge this gap, as multiple transcription factor families—including MYB, MADS-box, BEL1-like, APETALA2/ETHYLENE-RESPONSIVE FACTOR (AP2/ERF), zinc-finger, and hormone-responsive regulators—coordinate PME expression during seed development, organ growth, and fruit ripening (Ezquer et al., 2016; Xu et al., 2020; Liu et al., 2021; Wang et al., 2025b). Whether these developmental regulatory modules are also recruited during stress responses remains to be determined.

### Post-translational processing of PME precursors enables the timely generation of apoplastic danger signals

The absence of the N-terminal pro-region in type II PMEs suggests that these isoforms may reach their functional sites in a readily active form. In contrast, proper proteolytic processing of type I PMEs is critical for efficient plant responses to stress (Del Corpo et al., 2024). Type I PMEs contain a conserved processing motif, typically RRLL or RKLL, which can be recognized and cleaved by subtilases (SBTs) (Wolf et al., 2009; Coculo et al., 2023). In *A*. *thaliana* and other plant species, SBTs activate specific PME isoforms in response to developmental programs and environmental cues (Senechal et al., 2014; Meyer et al., 2016; Coculo et al., 2023). In *A. thaliana*, AtSBT3.3 and AtSBT3.5 modulates PME activity during *B. cinerea* infection, promoting rapid immune responses (Coculo et al., 2023). AtSBT3.3 cleaves the AtPME17 precursor, enabling the mature enzyme to de-methylesterify pectin and thereby contribute to defense responses against pathogens (Del Corpo et al., 2020). Notably, AtSBT3.3 is a key regulator of immune priming, a physiological state that enables faster and more robust activation of defense responses upon subsequent pathogen challenge (Ramirez et al., 2013; Conrath et al., 2015). Although AtSBT3.3 likely acts on multiple substrates, these findings raise the possibility that PME activation by SBTs may contribute to immune priming. Given the metabolic cost of defense, which imposes a growth–defense trade-off (He et al., 2022), timely proteolytic activation of PMEs upon pathogen challenge may help mitigate this constraint. The tight regulation of this system is further highlighted by the autocatalytic activation of SBTs, which are synthesized as pro-enzymes (Cedzich et al., 2009; Plattner et al., 2014).

Intriguingly, AtPME17 and AtSBT3.3 follow distinct yet coordinated trafficking routes to reach the apoplast. AtPME17 is delivered via the conventional secretory pathway, involving the endoplasmic reticulum and Golgi apparatus (Wang et al., 2017). By contrast, AtSBT3.3 is secreted through an unconventional secretory pathway, mediated by exocyst-positive organelles (EXPOs) (Ding et al., 2014; Coculo et al., 2023) (Figure 2). Moreover, in co-transformed *N. tabacum* cells, AtPME17 was detected in the apoplast before the arrival of AtSBT3.3. Such spatial and temporal segregation likely acts as a regulatory safeguard, preventing premature PME activation and untimely HG de-methylesterification during the secretory process. Evidence that microbial and insect PMEs lack the pro-region further corroborates this view (Markovic and Janecek, 2004). The absence of pectin-rich CWs in microbes and insects likely reduces the selective pressure for such regulatory control.

In addition to activating PMEs, *At*SBT3.5 also processes the SCOOP12 precursor, which gives rise to serine-rich endogenous peptides, thereby releasing the active phytocytokine SCOOP12 and promoting immune responses (Rzemieniewski and Stegmann, 2022; Yang et al., 2023). These findings support a model in which specific SBTs act as shared proteolytic switches that coordinate the activation of PMEs and phytocytokines, thereby enhancing plant immunity through both CW remodeling and peptide-mediated signaling (Del Corpo et al., 2024). How these pathways integrate with other elicitor-triggered signaling networks remains an open question.

### Dynamics of pectin methylesterification, cross-linking, and remodeling in cell wall integrity and protection

During diverse plant–microbe interactions, plants deploy different strategies to preserve CWI and restrict pathogen invasion (Seifert and Blaukopf, 2010; Rui and Dinneny, 2020). One important mechanism is the synthesis and maintenance of highly methylesterified HG, which limits the accessibility of pectin to microbial pectinolytic enzymes, such as polygalacturonases and pectate lyases, and reduces its susceptibility to degradation (Lionetti et al., 2012) (Figure 3A). Higher HG methylesterification correlates with increased resistance to *Pectobacterium carotovorum* in potato (McMillan et al., 1993; Marty et al., 1997), *Colletotrichum lindemuthianum* in bean (Boudart et al., 1998), *Ralstonia solanacearum* in *S. lycopersicum* (Wydra and Berl, 2006), and *Fusarium graminearum* in *Triticum aestivum* (Lionetti et al., 2015; Giancaspro et al., 2018). Early studies showed that overexpression of *AtPMEI1* and *AtPMEI2* increases pectin methylesterification and enhances resistance to *B. cinerea*, drawing attention to PMEIs as key regulators of pectin status during plant defense (Lionetti et al., 2007, 2014b). Subsequent studies identified PMEI isoforms that are induced and exploited in plant–pathogen interactions (Table 2). In particular, AtPMEI10, AtPMEI11, and AtPMEI12 modulate PME activity during *B. cinerea* infection (Lionetti et al., 2017). These inhibitors are induced to maintain CWI, especially during the later stages of infection, by preventing excessive pectin de-methylesterification that would otherwise promote pectin degradation by microbial pectinases. Consistent roles for PMEIs have been reported in other pathosystems. For example, *T. aestivum* plants expressing *AcPMEI* exhibit increased tolerance to *Bipolaris sorokiniana* and *F*. *graminearum* (Volpi et al., 2011; Tundo et al., 2016). In *Capsicum annuum*, silencing *CaPMEI1* increases susceptibility to *Xanthomonas campestris*, whereas its expression in *A. thaliana* enhances resistance to *Pseudomonas syringae* pv. *tomato* (An et al., 2008). Similarly, GhPMEI3 in *Gossypium hirsutum* inhibits GhPME2, and GhPME31 restricts infection by *Verticillium dahliae* (Liu et al., 2018; Zhang et al., 2022), whereas PvPMEI3 contributes to defense against *P*. *syringae* pv. *phaseolicola* in common bean (De La Rubia et al., 2024). PMEIs have also been implicated in defense against other types of biotic stress. For instance, *AtPMEI13* limits damage caused by the green peach aphid (*Myzus persicae*), restricting both feeding and colonization (Silva-Sanzana et al., 2019). Engineering PMEI expression therefore represents a promising biotechnological target for improving disease resistance across multiple pathosystems without typical growth–immunity trade-offs and may even increase biomass (Ferrari et al., 2008; Lionetti et al., 2010; Francocci et al., 2013).

**Table 2:** *Arabidopsis thaliana* PMEI isoforms mainly involved in biotic and abiotic stress responses.

Locus	PMEI isoform	Signal peptide	Transmembrane domain	Interacting organism(s) or abiotic stress	References
**Biotic stress**					
At1g48020	PMEI1	+	-	*B*. *cinerea*	(Lionetti et al., 2007)
At1g62760	PMEI10	+	-	*B*. *cinerea*	(Lionetti et al., 2017)
At3g17220	PMEI2	+	-	*B*. *cinerea* and *tobamoviruses*	(Lionetti et al., 2007, 2014a, 2014b)
At3g47380	PMEI11	+	+	*B. cinerea*	(Lionetti et al., 2017)
At5g46960	PMEI12	+	-	*B. cinerea*	(Lionetti et al., 2017)
At5g62360	PMEI13	+	-	*M*. *persicae*	(Silva-Sanzana et al., 2019)
**Abiotic stress**			type of abiotic stress		
At3g62820	PMEI18	+	-	drought	(Zhang et al., 2023)
At5g62360	PMEI13	+	-	cold	(Chen et al., 2018)

In addition to maintaining pectin in a protective state, plants can locally and robustly reinforce HG structure, especially beneath pathogen penetration sites, by forming CW appositions (papillae). Papilla formation involves coordinated CW remodeling, including pectin modification, polysaccharide-phenolic cross-linking, callose deposition, and the secretion of structural proteins (Pena et al., 2004; Rashid, 2016; Reem et al., 2016; Lionetti et al., 2017). Immunolabeling studies have detected the accumulation of low-methylesterified HG within papillae, suggesting localized PME activity (Vogel et al., 2002; Chowdhury et al., 2014). Plant PMEs generate contiguous blocks of de-methylesterified GalA residues, thereby spatially promoting Ca^2+^-mediated crosslinking between HG chains and strengthening the CW to counter pathogen penetration (Tibbits et al., 1998; Willats et al., 2001). In the classical egg-box model, Ca^2+^ ions coordinate adjacent HG chains to form ordered ionic bridges (Grant et al., 1973) (Figure 3B), structures that have recently been observed in CWs (Temple et al., 2022). Alternative arrangements such as the zipper model have also been proposed, in which Ca^2+^ bridges form primarily between opposing carboxylate groups of adjacent HG chains (Plazinski, 2011; Obomighie et al., 2025). AtPME17-mediated blockwise HG de-methylesterification reinforces the CW against *B. cinerea* infection (Del Corpo et al., 2020). Similarly, induction of *CjPME28* in *Camellia japonica* enhances CW fortification during herbivore attack (Dao et al., 2025). More broadly, PMEs have been implicated in the restoration of CWI in wound healing and graft union formation (Neubauer et al., 2012; Frey et al., 2024).

Apoplastic pH is dynamically modulated during plant-microbe interactions and strongly influences the outcome of PME-mediated pectin remodeling (Hua et al., 2018). Plants often trigger transient alkalinization of the apoplast during infection (Aziz et al., 2003), creating conditions that favor Ca^2+^-mediated pectin cross-linking. In contrast, necrotrophic fungi, such as *B*. *cinerea*, acidify host tissues through oxalic acid secretion, which chelates Ca^2+^ and destabilizes pectin networks (Manteau et al., 2003; Prusky and Yakoby, 2003). Acidic conditions may also shift PME activity from blockwise to random de-methylesterification, thereby increasing pectin susceptibility to pectinase attack (Hocq et al., 2024). Moreover, PME activity itself releases protons, potentially reinforcing local apoplastic acidification. Together, these processes highlight the dynamic interplay between plant- and pathogen-driven processes that compete to control apoplastic pH and CW stability.

### Contribution of PME activity to danger signal production and cell wall integrity sensing

In the absence of an adaptive immune system, plants rely on an innate immune network, whereby membrane-resident pattern-recognition receptors detect conserved pathogen- or microbe-associated molecular patterns (PAMPs/MAMPs) and activate pattern-triggered immunity (PTI) (DeFalco and Zipfel, 2021; Zhang et al., 2024a). PTI drives local and systemic defense responses, including the production of antimicrobial compounds, danger signaling, and CW remodeling to maintain CWI (Pogorelko et al., 2013; Lionetti et al., 2017; Swaminathan et al., 2022).

HG methylesterification patterns may encode spatiotemporal information within the CW, with PMEs acting as gatekeepers of pectin remodeling, integrity maintenance, and signaling (Figures 3C and 3D). Increasing evidence links PME activity to receptor systems that monitor apoplastic CW status. For example, the *A. thaliana* RESISTANCE TO FUSARIUM OXYSPORUM 1 (RFO1) protein acts as a sensor of HG methylesterification status during defense against *F. oxysporum* (Huerta et al., 2023) (Figure 3C). PME activity promotes the formation of negatively charged HG domains that serve as binding platforms for wall-associated remodeling proteins and wall sensors. In *A. thaliana*, de-methylesterified HG anchors the peroxidase PRX36 and Expansin-Like A1 (EXLA1), spatially modulating CW remodeling (Francoz et al., 2019; Men et al., 2026) (Figure 3D). De-methylesterified HG is a preferred substrate for the mechanosensor FERONIA (FER), a *Catharanthus rosesus* RECEPTOR-LIKE KINASE 1-LIKE (CrRLK1L) receptor kinase involved in several growth and immune signaling pathways (Guo et al., 2018; Cheung, 2024). FER forms signaling complexes with co-receptors, such as LORELEI-LIKE GPI-ANCHORED PROTEIN 1 (LLG1), and peptide ligands, such as RAPID ALKALINIZATION FACTORs (RALFs) (Stegmann et al., 2017; Gronnier et al., 2020; Abarca et al., 2021) (Figure 3D). De-methylesterified HG domains contribute to the spatial organization of FER-RALF signaling complexes at the cell surface (Rößling et al., 2024; Schoenaers et al., 2024). De-methylesterified HG also acts as a ligand for wall-associated kinase (WAK) receptors, which function in CWI sensing and OG perception (Decreux et al., 2006; Kohorn et al., 2014) (Figure 3D). Together, these findings support a model in which PME-driven pectin remodeling acts as a central hub integrating CW mechanics with receptor-mediated signaling at the cell surface.

PME activity can contribute to the generation of OGs, key pectin-derived damage-associated molecular patterns (DAMPs). These oligosaccharides are produced during microbial polygalacturonase-mediated HG degradation (Ferrari et al., 2013). Plants can modulate this process through the controlled inhibition of polygalacturonases by polygalacturonase-inhibiting proteins, which influence the size and elicitor activity of OGs (Xiao et al., 2024). HG methylesterification determines pectin susceptibility to enzymatic degradation (Le Cam et al., 1994; Lionetti, 2015), with fungal PMEs promoting random de-methylesterification patterns that enhance PG-mediated breakdown (Kohn et al., 1983; Limberg et al., 2000a, 2000b) (Figure 3D). Some plant PMEs also generate random patterns that increase pectin accessibility to pectinases in developmental contexts, although their contribution to OG production during infection remains unexplored (Catoire et al., 1998; Denes et al., 2000; Gallemí et al., 2025) (Figure 3). De-methylesterification of OGs by PMEs can also influence their elicitor activity (Osorio et al., 2008, 2011). Consistently, ectopic expression of the strawberry (*Fragaria* × *ananassa*) gene *FaPE1* in *Fragaria vesca* promotes the accumulation of bioactive OGs, leading to constitutive defense activation and enhanced resistance to *B*. *cinerea*. A complex mixture of OGs accumulates in the *A. thaliana*–*B. cinerea* interaction, including low-methylesterified oligomers, methylesterified fragments, and oxidized OGs (Benedetti et al., 2018; Voxeur et al., 2019). Recent work shows that RALF peptides bind de-methylesterified OGs, triggering phase separation and formation of OG–RALF–FER–LLG1 condensates that drive receptor endocytosis and initiate cell surface responses (Moussu et al., 2023; Liu et al., 2024; Rößling et al., 2024) (Figure 3D). Further linking PME activity to this context, recent evidence indicates that the *N. tabacum* LORELEI-like protein NtLLG4 acts as a receptor for PME isoforms (Weng et al., 2025). Although the functional relevance of these condensates in plant–microbe interactions remains to be defined, they highlight the potential structural role of PMEs in organizing and modulating receptor signaling at the cell surface.

PME activity also generates MeOH, a volatile compound that acts as a danger signal in intra- and inter-plant communication (Dorokhov et al., 2018) (Figure 3D). MeOH is rapidly released upon CW perturbation, particularly during wounding and herbivory, and modulates early signaling events including Ca^2+^ fluxes, membrane depolarization, and ethylene production (Von Dahl et al., 2006; Dorokhov et al., 2012a; Hann et al., 2014; Komarova et al., 2014). Moreover, MeOH emission from injured tissues can exert a “priming” effect on intact leaves of the same plant or on neighboring plants, preparing them for potential attack. Interestingly, MeOH may act primarily as a modulator, fine-tuning immune signaling rather than functioning as a strong elicitor, because it affects the amplitude of mitogen-activated protein kinase (MAPK) and reactive oxygen species (ROS) responses triggered by DAMPs/MAMPs (Tran et al., 2018). Studies in *Nicotiana attenuata* challenged with *Manduca sexta* and *Succisia pratensis* with *Euphydryas aurinia* indicate that PME-mediated MeOH release contributes to early defense activation, CW maintenance, and resistance to these herbivores (Peñuelas et al., 2005; Körner et al., 2009). However, contrasting evidence indicates that MeOH does not always enhance plant defense but can instead suppress specific defense traits, potentially increasing herbivore performance (Von Dahl et al., 2006). Moreover, MeOH-induced changes in plasmodesmata permeability may facilitate viral spread (Dorokhov et al., 2012a), highlighting its role as a context-dependent regulator. Finally, MeOH signaling has been hypothesized to extend beyond plant–plant communication, potentially acting as a cross-kingdom signal influencing animal responses, although its ecological relevance remains unclear (Dorokhov et al., 2012b). The mechanisms of MeOH perception and its physiological relevance remain poorly understood.

## Hijacking of PMEs by interacting organisms to modulate cell wall susceptibility

Although PMEs contribute to CW-based defense, interacting organisms can hijack specific PMEs to promote host colonization. These PME targets are often constitutively expressed in exposed tissues, offering an immediate entry point for pathogens and mutualists without requiring host transcriptional reprogramming. Plant PMEs can be manipulated to generate de-methylesterified domains targeted by microbial pectate lyases, thereby increasing CW susceptibility and facilitating host invasion. For instance, the *Ustilago maydis* effector Nge1 targets *Zea mays* ZmPMEI45 and ZmPMEI46, as well as the pro-region of ZmPME19 and ZmPME20, thereby releasing PME activity (Bhaskar et al., 2025). Similarly, in *A. thaliana*, AtPME3 is exploited by *B. cinerea* and *P*. *carotovorum* to promote pectin degradation and tissue maceration (Raiola et al., 2011). AtPME3 is also targeted during nematode infection, in which parasites induce extensive CW remodeling to establish feeding structures. The cyst nematode *Heterodera schachtii* secretes a cellulose-binding protein that interacts with AtPME3, likely promoting localized pectin de-methylesterification and wall loosening required for syncytium formation (Hewezi et al., 2008; Bohlmann and Sobczak, 2014; Pogorelko et al., 2019). The upregulation of *AtPMEPCRA* and *AtPME54* in *Meloidogyne javanica*-induced galls, together with the induction of soybean PME genes (*BE821923* and *AW30934*) during *Heterodera glycines* infection, suggests that this strategy is conserved across different nematode species. A similar strategy operates in other plant-pathogen interactions. AtPMEPCRA is exploited by *Plasmodiophora brassicae* to promote host cell death during clubroot disease (Stefanowicz et al., 2021). Moreover, in *G*. *hirsutum*, *Gh*PME36 promotes pectin de-methylesterification and increases carbohydrate availability, facilitating colonization by the leafminer *Liriomyza sativae* (Yang et al., 2024).

PMEs are also exploited by viruses to support viral movement. Movement proteins of several plant viruses, including *Tobacco mosaic virus* (*TMV*) and *Cauliflower mosaic virus* (*CaMV*), interact with PMEs to facilitate cell-to-cell spread (Chen and Citovsky, 2003; Stavolone and Lionetti, 2017). Because of the restricted size-exclusion limit of plasmodesmata, viral movement relies on movement protein-mediated modulation of plasmodesmata permeability and interactions with host factors (Carrington et al., 1996, Ghoshroy et al., 1997, Lazarowitz and Beachy, 1999). In this context, host PMEs contribute to plasmodesmata-mediated viral transport. An *N. tabacum* PME interacts with the *TMV* movement protein, and it is required for efficient viral spread (Chen et al., 2000). PMEs from *S. lycopersicum* and citrus interact with movement proteins of *turnip vein*-*clearing virus* (*TVCV*) and *CaMV*. Consistently, antisense suppression of a phloem-specific PME isoform in *N. tabacum* reduces the systemic movement of *TMV* (Chen and Citovsky, 2003). PME–movement protein interactions at plasmodesmata likely facilitate viral trafficking, although the underlying mechanism remains unresolved. Alternatively, movement protein interactions may modulate PME activity, thereby altering plasmodesmata permeability (Chen et al., 2000c). Consistently, overexpression of *AtPMEI2* in *A. thaliana* and *AcPMEI* in *N. tabacum* restricts both cell-to-cell and systemic spread of the tobamoviruses *Tobacco mosaic virus* (*TMV*) and *Turnip vein clearing virus* (*TVCV*) (Lionetti et al., 2014a, 2014b). Collectively, these findings identify PMEs as recurrent susceptibility factors targeted by viruses and other interacting organisms to reprogram host CW properties.

PME co-option is not restricted to pathogenic interactions but also occurs in beneficial associations. For example, AtPME30 can be exploited in the *A. thaliana*–*Bacillus subtilis* interaction to promote rhizobacterial colonization and enhance disease resistance (Lakshmanan et al., 2013). Similarly, in legume-rhizobial and actinorhizal symbioses, coordinated regulation of PMEs, PMEIs, and pectin-degrading enzymes supports infection and tissue remodeling (Hocher et al., 2011; Su et al., 2023).

## PMEs remodel the cell wall to mitigate abiotic stress

**Figure 4:**
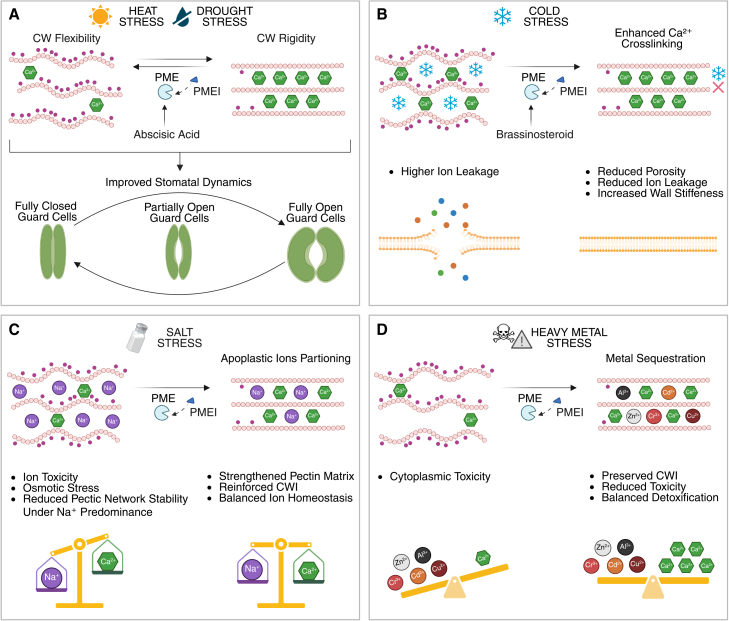
PME-mediated regulation of cell wall dynamics and ion homeostasis under abiotic stress. **(A)** Under heat and drought stress, abscisic acid signaling modulates the balance between PME and PMEI activities, fine-tuning guard cell CW flexibility and thereby supporting efficient stomatal dynamics. **(B)** Under cold stress, PME activity, regulated by PMEIs and brassinosteroid signaling, promotes Ca^2+^-mediated cross-linking, increasing CW stiffness while reducing porosity and ion leakage. **(C)** During salt stress, the PME–PMEI module balances the availability of free carboxyl groups on HG, promoting preferential Ca^2+^ cross-linking that strengthens the CW and mitigates Na^+^ toxicity. **(D)** Under heavy metal stress, PME activity increases the density of carboxyl groups on HG, enabling the sequestration of toxic ions, including Zn^2+^, Al^3+^, Cr^3+^, Cd^2+^, and Cu^2+^, within the CW. Colored spheres represent diffusible ions, including K^+^, Cl^−^, and Mg^2+^. PMEs: pectin methylesterases; PMEIs: PME inhibitors; HG: homogalacturonan; CW: cell wall.

Abiotic stresses severely affect plant growth and productivity by disrupting cellular homeostasis and impairing essential physiological processes (Jiang et al., 2025). Changes in CW mechanics and chemistry influence cell expansion, turgor maintenance, and signal transduction (Debnath et al., 2026). PMEs have emerged as key regulators of abiotic stress acclimation by modulating CW sensing and remodeling (Wu et al., 2018; Coculo and Lionetti, 2022) (Figure 4; Table 1). Compared with biotic interactions, the role of PMEs in CWI surveillance in abiotic stress responses remains less well defined. Nevertheless, core mechanisms related to CW sensing appear to be conserved. Elevated temperature and salinity promote phase separation involving de-methylesterified OG–RALF assemblies, recruiting FER–LLG1 into condensates and triggering receptor clustering and stress tolerance (Liu et al., 2024). Under salt stress, disruption of pectin–Ca^2+^ cross-links activates FER-dependent CWI signaling, Ca^2+^ bursts, MAPK cascades, and PME upregulation, thereby contributing to wall reinforcement and growth recovery (Feng et al., 2018). In addition, salinity rapidly induces PME activity upstream of the receptor-like kinases FER, HERCULES1 (HERK1), and THESEUS1 (THE1) (Gigli-Bisceglia et al., 2022), positioning PME-dependent pectin remodeling as an early event in salt stress perception. Receptors such as RLP44 and ERULUS, which sense pectin status and CW mechanics during development, may also participate in PME-dependent signaling under abiotic stress conditions, where CW mechanical constraints are altered (Wolf et al., 2014; Schoenaers et al., 2018). Beyond signaling, PMEs directly modulate CW physicochemical properties, including charge density, porosity, ion binding capacity, and mechanical properties such as stiffness and extensibility (Grant et al., 1973; Rondeau-Mouro et al., 2008; Caffall and Mohnen, 2009; Obomighie et al., 2025). These changes underpin key physiological processes, including stomatal dynamics, osmotic buffering, ion sequestration, and maintenance of CWI.

### Reprogramming CW dynamics during heat, drought, and cold stress

Climate change is increasing the co-occurrence of heat and drought, posing a combined threat to plant productivity and crop resilience. Although these stresses impose distinct physiological constraints, they converge on stomatal regulation as a central hub that balances water conservation and leaf cooling. During heat stress, plants must optimize stomatal aperture to sustain transpirational cooling while preventing excessive water loss. These stomatal dynamics are key determinants of thermotolerance. Stomatal density also contributes to longer-term acclimation (Chua and Lau, 2024). These responses integrate ABA signaling with CW mechanics of guard cells (Huang et al., 2016). In this context, PME-mediated pectin remodeling, together with apoplastic Ca^2+^, controls CW flexibility in guard cells (Wu and Jinn, 2010; Kan et al., 2023) (Figure 4A). AtPME34, which is expressed in guard cells, fine-tunes HG methylesterification required for stomatal function and heat tolerance (Huang et al., 2017; Wu et al., 2017). In addition to regulating stomatal dynamics, AtPME53 and AtPME12 regulate stomatal pore formation and density under heat stress, further linking pectin remodeling to ABA pathways (Wu et al., 2022, 2025). Drought stress drives stomatal adjustments to maintain water status (Osakabe et al., 2014). PME-mediated pectin remodeling also contributes to drought responses (Sun et al., 2025) (Figure 4A). In *A. thaliana*, AtPME6-mediated pectin de-methylesterification supports proper stomatal function during drought stress (Amsbury et al., 2016). Overexpression of *PtoPME35* in *Populus tomentosa* and *AcPME2* in *Actinidia chinensis* enhances drought tolerance by increasing de-methylesterified pectin and Ca^2+^-pectate interactions, thereby improving stomatal control (Yang et al., 2020; Zhu et al., 2024b). Drought responses also rely on coordinated PME–PMEI activity (An et al., 2008; Cheng et al., 2022; Zhang et al., 2024b). AtPME31 and its cognate inhibitor AtPMEI18 regulate optimal CW mechanics of guard cells in *A. thaliana* during drought conditions (Zhang et al., 2023). Together, these findings indicate that PME-dependent pectin remodeling dynamically regulates guard cell CW mechanics through similar mechanisms under heat and drought stress (Figure 4A). PME activity is tightly tuned by PMEIs to maintain a highly methylesterified, elastic wall during stomatal movement. PME activation, in turn, promotes Ca^2+^-mediated cross-linking and wall stiffening when movement ceases. This mechanism enables stomatal dynamics through stress-dependent shifts between wall flexibility and mechanical reinforcement. However, the biomechanical consequences of HG methylesterification cannot be generalized across different stomatal cell types. In commelinid grasses, which possess dumbbell-shaped guard cells, methylesterified HG accumulates at the polar ends of guard cells, where this distribution is associated with mechanically resistant wall domains that constrain stomatal opening (Zhang et al., 2026).

Cold stress primarily affects membrane stability but also induces extensive CW remodeling, including changes in wall thickness, ultrastructure, porosity, and polysaccharide composition (Campos et al., 2003; Solecka et al., 2008; Takahashi et al., 2021). Cold-acclimated tissues exhibit increased PME expression and accumulate low-methylesterified HG, which promotes Ca^2+^-mediated crosslinking and the formation of a semi-rigid pectate network (Baldwin et al., 2014; Lin et al., 2025) (Figure 4B). In *A. thaliana*, AtPME41 is required for cold-induced PME activity (Qu et al., 2011). *pme41* mutants show increased ion leakage without changes in membrane lipid peroxidation, indicating that cold tolerance relies on CWI rather than membrane stability. Brassinosteroid regulation of *AtPME41* further links hormonal signaling to pectin remodeling during cold acclimation. The involvement of AtPMEI13 in freezing tolerance further highlights the importance of PME regulation under abiotic stress (Chen et al., 2018; Zhang et al., 2024b). Thus, cold stress induces PME expression and drives a shift toward low-methylesterified HG that promotes Ca^2+^-mediated cross-linking and the formation of a semi-rigid pectate network, thereby reinforcing CWI and limiting ion leakage (Figure 4B). The persistence of these Ca^2+^-mediated cross-links beyond the acclimation phase further suggests that PME-driven remodeling may establish a form of “CW structural memory,” whereby enduring changes in HG organization and CW mechanics influence subsequent responses to freezing stress (Solecka et al., 2008; Shikata et al., 2026).

### Tuning ion homeostasis through pectin remodeling under salt stress

Salt stress disrupts cellular homeostasis through the combined effects of osmotic imbalance, ionic toxicity, and altered Ca^2+^ availability, ultimately destabilizing the pectin network and impairing CW mechanics. PME-mediated de-methylesterification represents a key adaptive response (Figure 4C). Genetic evidence indicates that AtPME31 and AtPME47 contribute to salt tolerance, as their loss-of-function mutants are hypersensitive to NaCl (Yan et al., 2018; Zhou et al., 2024). Notably, AtPME31 exhibits low PME activity *in vitro* and atypical subcellular localization, suggesting that its contribution to salt tolerance may involve non-canonical functions beyond direct pectin remodeling, possibly linked to lipid metabolism (Dedeurwaerder et al., 2008; Hamade et al., 2025). Overexpression of *NtPME043* enhances root growth under high-salt conditions (Sun et al., 2022). Collectively, these findings support a model in which PME activity increases the availability of carboxyl groups on HG for cation binding, thereby influencing apoplastic Ca^2+^/Na^+^ partitioning and ion buffering capacity (Pilling et al., 2000, 2004). Preferential Ca^2+^-mediated cross-linking stabilizes the pectic network and limits Na^+^ interference, thereby preserving wall hydration and maintaining CWI under salinity. PMEI-mediated regulation further fine-tunes this balance (Jithesh et al., 2012; Chen et al., 2018) (Figure 4C).

### PME-dependent control of pectin charge regulates ion homeostasis and metal sequestration

Beyond salinity, PME-dependent modulation of pectin charge represents a general mechanism regulating ion binding, sequestration, and distribution under diverse abiotic stresses. Heavy-metal stress imposes constraints that often converge on the CW. By increasing the density of free carboxyl groups on HG, PME-mediated de-methylesterification modulates metal binding, mobility, and toxicity. Depending on the context, this remodeling can either protect the cytoplasm by immobilizing toxic ions in the CW or exacerbate toxicity by altering CW porosity, elasticity, or Ca^2+^-mediated cross-linking. Evidence from *A. thaliana* illustrates this context-dependent role (Figure 4D). Overexpression of *AtPME3* leads to hypersensitivity to Zn^2+^, indicating that excessive de-methylesterification enhances metal binding and perturbs ionic homeostasis (Weber et al., 2013). Responses to aluminum (Al^3+^) stress appear more tightly regulated: AtPME46 limits Al^3+^ binding and alleviates root growth inhibition, whereas AtPME17 and AtPME54 are repressed upon Al^3+^ exposure, suggesting that plants restrict excessive wall negative charge to prevent excessive Al^3+^ accumulation (Geng et al., 2017). In rice, overexpression of *OsPME14* increases Al^3+^ retention and root growth inhibition, likely through Ca^2+^ displacement from de-methylesterified pectin (Yang et al., 2013). The Al-responsive *CsPME6* gene in *Camellia sinensis* is integrated into WRKY-dependent transcriptional networks (Huang et al., 2022, 2025). Similarly, increased de-methylesterification enhances binding and sequestration of other metals, including chromium (Cr^3+^), cadmium (Cd^2+^), and copper (Cu^2+^) (Paynel et al., 2009; Zhang et al., 2021; Ullah et al., 2023; Yu et al., 2023). In rice, coordinated regulation of OsPME14 and its inhibitor OsPMEI6 modulates the Cu^2+^-binding capacity of pectin (Wang et al., 2025a).

Beyond detoxification, PME-dependent control of pectin charge also regulates nutrient availability. In the *S. lycopersicum Colorless nonripening* (*Cnr*) mutant, reduced pectin methylesterification enhances iron (Fe) binding and immobilization, triggering deficiency responses despite sufficient external Fe supply (Zhu et al., 2024a). Similarly, under phosphorus (P) deficiency, increased de-methylesterification promotes both P remobilization and Cd^2+^ association with the wall (Qi et al., 2022; Li et al., 2024). PMEs also influence post-harvest physiology, during which tissues experience mechanical injury, oxidative bursts, and dehydration. In *Manihot esculenta*, *MePME1* induction correlates with susceptibility to post-harvest deterioration, whereas limited induction in resistant varieties helps maintain CWI and delays tissue collapse (Wang et al., 2023).

## Tracking PME activity in plant tissues: From biochemical assays to imaging

A wide range of analytical approaches has been developed to detect and quantify PME activity, largely based on changes in pectin charge or the release of reaction products. The PECTOPLATE assay is widely used to monitor PME activity under stress conditions (Lionetti, 2015). This approach combines a ruthenium red-based gel readout, which selectively detects de-methylesterified pectin, with high-performance anion-exchange chromatography coupled to pulsed amperometric detection (HPAEC-PAD), enabling the simultaneous assessment of PME and pectinase activities as well as OG release. Additional colorimetric methods rely on pH changes or the titration of carboxyl groups released during de-methylesterification, providing simple and low-cost approaches, although they have limited sensitivity and are subject to potential interference from organic acids.

Quantification of PME activity can also be achieved indirectly by measuring MeOH release. Alcohol oxidase-based enzymatic assays coupled to colorimetric detection have largely replaced earlier chemical methods, offering improved specificity, sensitivity, and compatibility with high-throughput formats (Anthon and Barrett, 2004). Complementary approaches, such as headspace gas chromatography (GC) or GC-MS, enable accurate quantification of MeOH emissions from plant tissues, allowing the *in vivo* assessment of PME activity, while proton-transfer-reaction (PTR)-MS allows real-time monitoring of MeOH release dynamics (Nemecekmarshall et al., 1995; Fall and Benson, 1996; Dorokhov et al., 2012a).

Complementary structural techniques provide detailed information on pectin methylesterification status. Fourier-transform infrared (FT-IR) spectroscopy is widely used because it requires minimal sample preparation and can rapidly estimate the degree of esterification without chemical pretreatment (Manrique and Lajolo, 2002). Chromatographic approaches, such as high performance liquid chromatography (HPLC) and GC, enable quantitative determination of the degree of methyl- and acetyl-esterification following pectin saponification and the release of MeOH and acetic acid (Levigne et al., 2002; Savary and Nuñez, 2003). Proton nuclear magnetic resonance (^1^H-NMR) spectroscopy enables simultaneous, high-resolution assessment of methyl- and acetyl-esterification (Grasdalen et al., 1988). Mass spectrometry-based approaches provide high-resolution insights into pectin structure and remodeling. Oligosaccharide mass profiling (OLIMP) coupled with matrix-assisted laser desorption/ionization time-of-flight (MALDI-TOF) MS has proven particularly powerful for detecting pathogen-induced changes in pectin methylesterification and for linking alterations in esterification patterns to modifications in CW architecture (Lionetti et al., 2007).

Recent advances in molecular recognition and imaging technologies have expanded the analytical toolbox for probing pectin methylesterification. Immunological methods based on monoclonal antibodies (e.g., JIM5, JIM7, and the LM series), combined with platforms such as comprehensive microarray polymer profiling (CoMPP), enable the *in situ* and semi-quantitative profiling of HG epitopes with defined methylesterification patterns (Moller et al., 2008; Pattathil et al., 2010, 2012; Kračun et al., 2017). These approaches have been used to visualize pathogen-induced shifts in pectin structure, including changes associated with *B. cinerea* infection (Lionetti et al., 2017). Complementary low-molecular-weight probes, including propidium iodide, Coriphosphine O, chitosan oligosaccharides (COS488 and COS647), and OG-based probes (OG488), allow the *in situ* detection of de-methylesterified HG domains with improved tissue penetration and compatibility with live imaging (Harenčár et al., 2025). A super-resolution approach, 3D direct stochastic optical reconstruction microscopy (3D-dSTORM), enables nanoscale *in muro* visualization of pectin nanostructure, including the spatial organization of methylesterified and de-methylesterified HG domains and their association with CW-associated proteins (Moussu et al., 2023; Jia et al., 2026).

In summary, PME activity dynamically integrates HG remodeling with CW-associated signaling across diverse environmental conditions in a spatiotemporally controlled manner and is tightly regulated by PMEIs and SBTs. PME activity thereby establishes a functional interface that integrates mechanical reinforcement, receptor organization, and danger signal generation. Rather than acting through a linear pathway, PME activity operates as a context-dependent switch, in which Ca^2+^ availability, apoplastic pH, and de-methylesterification patterns collectively determine the balance between wall stiffening and loosening, as well as between resistance and susceptibility. Ultimately, PME function should be viewed as part of a dynamic and competitive process at the plant surface, where plant- and microbe-derived activities converge to shape pectin structure and downstream signaling outputs. PMEs therefore emerge as key determinants of abiotic stress adaptation, enabling plants to fine-tune CW mechanics along a flexibility-rigidity continuum while simultaneously coordinating signaling pathways required for stress resilience. Despite these advances, significant knowledge gaps persist. Although the expansion of PME gene families has been documented, the *in vivo* roles and functional specialization of individual isoforms remain largely unresolved. Dissecting the relative roles of type I and type II PMEs will be essential for determining how distinct regulatory architectures translate into specific patterns of pectin remodeling across developmental and stress contexts. In parallel, pectin remodeling during biotic interactions results from the dynamic and potentially competitive actions of both plant- and microbe-derived PMEs, although their interplay in shaping HG structure remains poorly understood.

Acting in a highly context-dependent manner, PMEs generate distinct de-methylesterification patterns. However, determining how these patterns arise and are precisely controlled in space and time through secretion, proteolytic activation, and the CW physicochemical properties remains a major challenge, particularly in the context of stress responses. Importantly, the enzymatic activity of many PME isoforms has yet to be experimentally validated, limiting functional interpretation. Another unresolved aspect concerns substrate specificity. Although the rhamnogalacturonan II backbone can also be methylesterified (O’Neill et al., 2020), direct evidence for PME isoforms specific for the de-methylesterification of rhamnogalacturonan is still lacking.

How PME-driven changes in CW stiffness, charge density, and Ca^2+^–pectate organization are integrated with diverse stress signals remains to be clarified. Likewise, the extent to which de-methylesterified HG and/or OGs control the assembly, dynamics, and endocytosis of receptor complexes such as FER–RALF is only partially understood. More broadly, how specific methylesterification patterns are decoded into distinct cellular signaling outputs remains a central open question.

From an applied perspective, PME-mediated responses represent promising targets for enhancing crop stress resilience. This will require the development of genetic and biotechnological strategies to achieve spatiotemporal and isoform-specific control of PME activity, including its targeted localization at sites of CW remodeling such as pathogen penetration zones. These approaches may include the overexpression of PMEIs, targeted manipulation of stress-responsive transcription factors, and structure-guided engineering of PME–PMEI and PME–SBT interactions, with the goal of enabling precise modulation of pectin methylesterification, optimization of CW mechanics, and tuning of CWI sensing under stress conditions.

## Funding

V.L. is supported by 10.13039/501100004271Sapienza University of Rome, grants RG12419107C1C64B and CHE12418E16493B1F, by 10.13039/501100000780European Union “NextGenerationEU” programme–PNRR–Ecosistemi dell’Innovazione Project ECS 0000024 Rome Technopole: CUP B83C22002820006 and Project ECS 00000043 Consorzio iNEST: CUP B43C22000450006, by the Italian Ministry of Education, University and Research (MUR) through the projects PRIN2022 XyWall: Cell wall determinants in plant resistance to Xylella 2022F8BZMX CUP B53C24007450001 and “REACH-XY”: CUP B93C22001920001, and by the HORIZON-CL6-2024-ZEROPOLLUTION-02-2 project OLinWASTE-101181402 CUP B83C24008790006.

## Acknowledgments

The authors apologize to colleagues whose studies were not cited owing to space limitations. Figures were created with Biorender.com.

## Author contributions

Conceptualization, V.L.; writing – original draft, G.P., G.C, D.C., and V.L.; writing – review and editing, G.P., D.C., and V.L. funding acquisition, V.L.

## References

[bib1] Abarca A., Franck C.M., Zipfel C. (2021). Family-wide evaluation of RAPID ALKALINIZATION FACTOR peptides. Plant Physiol..

[bib2] Amsbury S., Hunt L., Elhaddad N., Baillie A., Lundgren M., Verhertbruggen Y., Scheller H.V., Knox J.P., Fleming A.J., Gray J.E. (2016). Stomatal Function Requires Pectin De-methyl-esterification of the Guard Cell Wall. Curr. Biol..

[bib3] An S.H., Sohn K.H., Choi H.W., Hwang I.S., Lee S.C., Hwang B.K. (2008). Pepper pectin methylesterase inhibitor protein CaPMEI1 is required for antifungal activity, basal disease resistance and abiotic stress tolerance. Planta.

[bib4] Anthon G.E., Barrett D.M. (2004). Comparison of Three Colorimetric Reagents in the Determination of Methanol with Alcohol Oxidase. Application to the Assay of Pectin Methylesterase. J. Agric. Food Chem..

[bib5] Atmodjo M.A., Sakuragi Y., Zhu X., Burrell A.J., Mohanty S.S., Atwood J.A., Orlando R., Scheller H.V., Mohnen D. (2011). Galacturonosyltransferase (GAUT)1 and GAUT7 are the core of a plant cell wall pectin biosynthetic homogalacturonan: galacturonosyltransferase complex. Proc. Natl. Acad. Sci. USA.

[bib6] Aziz A., Poinssot B., Daire X., Adrian M., Bézier A., Lambert B., Joubert J.M., Pugin A. (2003). Laminarin elicits defense responses in grapevine and induces protection against Botrytis cinerea and Plasmopara viticola. Mol. Plant Microbe Interact..

[bib7] Bacete L., Hamann T. (2020). The Role of Mechanoperception in Plant Cell Wall Integrity Maintenance. Plants.

[bib8] Baez L.A., Tichá T., Hamann T. (2022). Cell wall integrity regulation across plant species. Plant Mol. Biol..

[bib9] Baldwin L., Domon J.-M., Klimek J.F., Fournet F., Sellier H., Gillet F., Pelloux J., Lejeune-Hénaut I., Carpita N.C., Rayon C. (2014). Structural alteration of cell wall pectins accompanies pea development in response to cold. Phytochemistry.

[bib10] Bellincampi D., Cervone F., Lionetti V. (2014). Plant cell wall dynamics and wall-related susceptibility in plant-pathogen interactions. Front.Plant Sci.

[bib11] Benedetti M., Verrascina I., Pontiggia D., Locci F., Mattei B., De Lorenzo G., Cervone F. (2018). Four Arabidopsis berberine bridge enzyme-like proteins are specific oxidases that inactivate the elicitor-active oligogalacturonides. Plant J..

[bib12] Bethke G., Grundman R.E., Sreekanta S., Truman W., Katagiri F., Glazebrook J. (2014). Arabidopsis PECTIN METHYLESTERASEs Contribute to Immunity against Pseudomonas syringae. Plant Physiol..

[bib13] Bethke G., Thao A., Xiong G., Li B., Soltis N.E., Hatsugai N., Hillmer R.A., Katagiri F., Kliebenstein D.J., Pauly M., Glazebrook J. (2016). Pectin Biosynthesis Is Critical for Cell Wall Integrity and Immunity in Arabidopsis thaliana. Plant Cell.

[bib14] Bhandari D.D., Kim S.-J., Brandizzi F. (2025). Fortifying the frontier: cell wall modifications during plant immunity. Curr. Opin. Plant Biol..

[bib15] Bhaskar C., Chau M.-Q., Tsai W.-L., Teh O.-K., Ma L.-S. (2025). N-glycosylation Enables Smut Fungal Nge1 Orthologs to Prevent the Escape of Maize Evolved-PMEIs. bioRxiv.

[bib16] Bohlmann H., Sobczak M. (2014). The plant cell wall in the feeding sites of cyst nematodes. Front. Plant Sci..

[bib17] Bosch M., Hepler P.K. (2005). Pectin Methylesterases and Pectin Dynamics in Pollen Tubes. Plant Cell.

[bib18] Boudart G., Lafitte C., Barthe J.P., Frasez D., Esquerré-Tugayé M.T. (1998). Differential elicitation of defense responses by pectic fragments in bean seedlings. Planta.

[bib19] Caffall K.H., Mohnen D. (2009). The structure, function, and biosynthesis of plant cell wall pectic polysaccharides. Carbohydr. Res..

[bib100] Cam B.L., Massiot P., Campion C., Rouxel F. (1994). Susceptibility of carrot cultivars to Mycocentrospora acerina and the structure of cell wall polysaccharides. Physiol. Mol. Plant Pathol..

[bib20] Campos P.S., Quartin V.n., Nunes M.A., Ramalho J.c. (2003). Electrolyte leakage and lipid degradation account for cold sensitivity in leaves of*Coffea* sp. plants. J. Plant Physiol..

[bib21] Canut H., Albenne C., Jamet E. (2016). Post-translational modifications of plant cell wall proteins and peptides: A survey from a proteomics point of view. Biochim. Biophys. Acta.

[bib237] Carrington J.C., Kasschau K.D., Mahajan S.K., Schaad M.C. (1996). Cell-to-cell and long-distance transport of viruses in plants. Plant Cell.

[bib22] Catoire L., Pierron M., Morvan C., du Penhoat C.H., Goldberg R. (1998). Investigation of the action patterns of pectinmethylesterase isoforms through kinetic analyses and NMR spectroscopy. Implications In cell wall expansion. J. Biol. Chem..

[bib23] Cedzich A., Huttenlocher F., Kuhn B.M., Pfannstiel J., Gabler L., Stintzi A., Schaller A. (2009). The protease-associated domain and C-terminal extension are required for zymogen processing, sorting within the secretory pathway, and activity of tomato subtilase 3 (SlSBT3). J. Biol. Chem..

[bib24] Chen M.-H., Citovsky V. (2003). Systemic movement of a tobamovirus requires host cell pectin methylesterase. Plant J..

[bib25] Chen J., Chen X., Zhang Q., Zhang Y., Ou X., An L., Feng H., Zhao Z. (2018). A cold-induced pectin methyl-esterase inhibitor gene contributes negatively to freezing tolerance but positively to salt tolerance in Arabidopsis. J. Plant Physiol..

[bib234] Chen M.H., Sheng J., Hind G., Handa A.K., Citovsky V. (2000). Interaction between the tobacco mosaic virus movement protein and host cell pectin methylesterases is required for viral cell-to-cell movement. EMBO J..

[bib26] Cheng S.-S., Ku Y.-S., Cheung M.-Y., Lam H.-M. (2022). Identification of stably expressed reference genes for expression studies in Arabidopsis thaliana using mass spectrometry-based label-free quantification. Front. Plant Sci..

[bib27] Cheung A.Y. (2024). FERONIA: A Receptor Kinase at the Core of a Global Signaling Network. Annu. Rev. Plant Biol..

[bib28] Chowdhury J., Henderson M., Schweizer P., Burton R.A., Fincher G.B., Little A. (2014). Differential accumulation of callose, arabinoxylan and cellulose in nonpenetrated versus penetrated papillae on leaves of barley infected with Blumeria graminis f. sp. hordei. New Phytol..

[bib29] Chua L.C., Lau O.S. (2024). Stomatal development in the changing climate. Development.

[bib30] Ciardiello M.A., D’Avino R., Amoresano A., Tuppo L., Carpentieri A., Carratore V., Tamburrini M., Giovane A., Pucci P., Camardella L. (2008). The peculiar structural features of kiwi fruit pectin methylesterase: amino acid sequence, oligosaccharides structure, and modeling of the interaction with its natural proteinaceous inhibitor. Proteins.

[bib31] Coculo D., Lionetti V. (2022). The Plant Invertase/Pectin Methylesterase Inhibitor Superfamily. Front. Plant Sci..

[bib32] Coculo D., Del Corpo D., Martínez M.O., Vera P., Piro G., De Caroli M., Lionetti V. (2023). Arabidopsis subtilases promote defense-related pectin methylesterase activity and robust immune responses to botrytis infection. Plant Physiol. Biochem..

[bib33] Conrath U., Beckers G.J.M., Langenbach C.J.G., Jaskiewicz M.R. (2015). Priming for Enhanced Defense. Annu. Rev. Phytopathol..

[bib34] Dao M., Li J., Wang M., Wang X., Zhang H., Chen L., Wu T. (2025). Transcriptomic analysis of Camellia japonica to scale insects infestation and functional characterization of pectin methylesterase gene CjPME28 and polygalacturonase gene CjPG1. Plant Cell Rep..

[bib35] De La Rubia A.G., Largo-Gosens A., Yusta R., Sepúlveda-Orellana P., Riveros A., Centeno M.L., Sanhueza D., Meneses C., Saez-Aguayo S., García-Angulo P. (2024). A novel pectin methylesterase inhibitor, PMEI3, in common bean suggests a key role of pectin methylesterification in *Pseudomonas* resistance. J. Exp. Bot..

[bib36] Debnath J., Morton R.N., Engelsdorf T., Gigli-Bisceglia N. (2026). Plant Cell Wall Remodeling and Peptide Signaling Under Abiotic and Biotic Stress. Plant Commun..

[bib37] Decreux A., Thomas A., Spies B., Brasseur R., Cutsem P., Messiaen J. (2006). In vitro characterization of the homogalacturonan-binding domain of the wall-associated kinase WAK1 using site-directed mutagenesis. Phytochemistry.

[bib38] Dedeurwaerder S., Menu-Bouaouiche L., Mareck A., Lerouge P., Guerineau F. (2008). Activity of an atypical Arabidopsis thaliana pectin methylesterase. Planta.

[bib39] DeFalco T.A., Zipfel C. (2021). Molecular mechanisms of early plant pattern-triggered immune signaling. Mol. Cell.

[bib40] Del Corpo D., Fullone M.R., Miele R., Lafond M., Pontiggia D., Grisel S., Kieffer-Jaquinod S., Giardina T., Bellincampi D., Lionetti V. (2020). AtPME17 is a functional Arabidopsis thaliana pectin methylesterase regulated by its PRO region that triggers PME activity in the resistance to Botrytis cinerea. Mol. Plant Pathol..

[bib41] Del Corpo D., Coculo D., Greco M., De Lorenzo G., Lionetti V. (2024). Pull the fuzes: Processing protein precursors to generate apoplastic danger signals for triggering plant immunity. Plant Commun..

[bib42] Delmer D., Dixon R.A., Keegstra K., Mohnen D. (2024). The plant cell wall—dynamic, strong, and adaptable—is a natural shapeshifter. Plant Cell.

[bib43] Denès J.M., Baron A., Renard C.M.G.C., Péan C., Drilleau J.F. (2000). Different action patterns for apple pectin methylesterase at pH 7.0 and 4.5. Carbohydr. Res..

[bib44] Di Matteo A., Giovane A., Raiola A., Camardella L., Bonivento D., De Lorenzo G., Cervone F., Bellincampi D., Tsernoglou D. (2005). Structural Basis for the Interaction between Pectin Methylesterase and a Specific Inhibitor Protein. Plant Cell.

[bib45] Ding Y., Robinson D.G., Jiang L. (2014). Unconventional protein secretion (UPS) pathways in plants. Curr. Opin. Cell Biol..

[bib46] Dorokhov Y.L., Komarova T.V., Petrunia I.V., Frolova O.Y., Pozdyshev D.V., Gleba Y.Y. (2012). Airborne Signals from a Wounded Leaf Facilitate Viral Spreading and Induce Antibacterial Resistance in Neighboring Plants. PLoS Pathog..

[bib47] Dorokhov Y.L., Komarova T.V., Petrunia I.V., Kosorukov V.S., Zinovkin R.A., Shindyapina A.V., Frolova O.Y., Gleba Y.Y. (2012). Methanol May Function as a Cross-Kingdom Signal. PLoS One.

[bib48] Dorokhov Y.L., Sheshukova E.V., Komarova T.V. (2018). Methanol in Plant Life. Front. Plant Sci..

[bib49] Du J., Anderson C.T., Xiao C. (2022). Dynamics of pectic homogalacturonan in cellular morphogenesis and adhesion, wall integrity sensing and plant development. Nat. Plants.

[bib50] Engelsdorf T., Gigli-Bisceglia N., Veerabagu M., McKenna J.F., Vaahtera L., Augstein F., Van der Does D., Zipfel C., Hamann T. (2018). The plant cell wall integrity maintenance and immune signaling systems cooperate to control stress responses in Arabidopsis thaliana. Sci. Signal..

[bib51] Engle K.A., Amos R.A., Yang J., Glushka J., Atmodjo M.A., Tan L., Huang C., Moremen K.W., Mohnen D. (2022). Multiple Arabidopsis galacturonosyltransferases synthesize polymeric homogalacturonan by oligosaccharide acceptor-dependent or *de novo* synthesis. Plant J..

[bib52] Eulgem T., Rushton P.J., Robatzek S., Somssich I.E. (2000). The WRKY superfamily of plant transcription factors. Trends Plant Sci..

[bib53] Ezquer I., Mizzotti C., Nguema-Ona E., Gotté M., Beauzamy L., Viana V.E., Dubrulle N., Costa de Oliveira A., Caporali E., Koroney A.-S. (2016). The Developmental Regulator SEEDSTICK Controls Structural and Mechanical Properties of the Arabidopsis Seed Coat. Plant Cell.

[bib54] Fall R., Benson A.A. (1996). Leaf methanol — the simplest natural product from plants. Trends Plant Sci..

[bib55] Feng W., Kita D., Peaucelle A., Cartwright H.N., Doan V., Duan Q., Liu M.-C., Maman J., Steinhorst L., Schmitz-Thom I. (2018). The FERONIA Receptor Kinase Maintains Cell-Wall Integrity during Salt Stress through Ca2+ Signaling. Curr. Biol..

[bib56] Ferrari S., Galletti R., Pontiggia D., Manfredini C., Lionetti V., Bellincampi D., Cervone F., De Lorenzo G. (2008). Transgenic Expression of a Fungal endo-Polygalacturonase Increases Plant Resistance to Pathogens and Reduces Auxin Sensitivity. Plant Physiology.

[bib57] Ferrari S., Savatin D.V., Sicilia F., Gramegna G., Cervone F., De Lorenzo G. (2013). Oligogalacturonides: plant damage-associated molecular patterns and regulators of growth and development. Front.Plant Sci..

[bib58] Francocci F., Bastianelli E., Lionetti V., Ferrari S., De Lorenzo G., Bellincampi D., Cervone F. (2013). Analysis of pectin mutants and natural accessions of Arabidopsis highlights the impact of de-methyl-esterified homogalacturonan on tissue saccharification. Biotechnol. Biofuels.

[bib59] Francoz E., Ranocha P., Le Ru A., Martinez Y., Fourquaux I., Jauneau A., Dunand C., Burlat V. (2019). Pectin Demethylesterification Generates Platforms that Anchor Peroxidases to Remodel Plant Cell Wall Domains. Dev. Cell.

[bib60] Frey C., Saez-Aguayo S., Encina A., Acebes J.L. (2024). Deepening the Role of Pectin in the Tissue Assembly Process During Tomato Grafting. Plants.

[bib61] Gallemí M., Montesinos J.C., Zarevski N., Pribyl J., Skládal P., Hannezo E., Benková E. (2025). Dual role of pectin methyl esterase activity in the regulation of plant cell wall biophysical properties. Front. Plant Sci..

[bib62] Geng X., Horst W.J., Golz J.F., Lee J.E., Ding Z., Yang Z. (2017). LEUNIG_HOMOLOG transcriptional co-repressor mediates aluminium sensitivity through PECTIN METHYLESTERASE 46-modulated root cell wall pectin methylesterification in Arabidopsis. Plant J..

[bib235] Ghoshroy S., Lartey R., Sheng J., Citovsky V. (1997). Transport of proteins and nucleic acids through plasmodesmata. Annu. Rev. Plant Physiol. Plant Mol. Biol..

[bib63] Giancaspro A., Lionetti V., Giove S.L., Zito D., Fabri E., Reem N., Zabotina O.A., De Angelis E., Monaci L., Bellincampi D., Gadaleta A. (2018). Cell wall features transferred from common into durum wheat to improve Fusarium Head Blight resistance. Plant Sci..

[bib64] Gigli-Bisceglia N., van Zelm E., Huo W., Lamers J., Testerink C. (2022). Arabidopsis root responses to salinity depend on pectin modification and cell wall sensing. Development.

[bib65] Grant G.T., Morris E.R., Rees D.A., Smith P.J.C., Thom D. (1973). Biological interactions between polysaccharides and divalent cations: The egg-box model. FEBS (Fed. Eur. Biochem. Soc.) Lett..

[bib66] Grasdalen H., Einar Bakøy O., Larsen B. (1988). Determination of the degree of esterification and the distribution of methylated and free carboxyl groups in pectins by 1H-n.m.r. spectroscopy. Carbohydr. Res..

[bib67] Gronnier J., Franck C.M., Stegmann M., DeFalco T.A., Cifuentes A.A., Dünser K., Lin W., Yang Z., Kleine-Vehn J., Ringli C. (2020). FERONIA regulates FLS2 plasma membrane nanoscale dynamics to modulate plant immune signaling. bioRxiv.

[bib68] Guo H., Nolan T.M., Song G., Liu S., Xie Z., Chen J., Schnable P.S., Walley J.W., Yin Y. (2018). FERONIA Receptor Kinase Contributes to Plant Immunity by Suppressing Jasmonic Acid Signaling in Arabidopsis thaliana. Curr. Biol..

[bib69] Hamade S., Traver M.S., Bartel B. (2025). The Atypical Pectin Methylesterase Family Member PME31 Promotes Seedling Lipid Droplet Utilization. Plant Direct.

[bib70] Hann C.T., Bequette C.J., Dombrowski J.E., Stratmann J.W. (2014). Methanol and ethanol modulate responses to danger- and microbe-associated molecular patterns. Front. Plant Sci..

[bib71] Harenčár Ľ., Heldesová K., Stratilová B., Kumar A., Mravec J. (2025). Probing homogalacturonan *in situ:* A comprehensive review of available molecular recognition tools. Int. J. Biol. Macromol..

[bib72] He Z., Webster S., He S.Y. (2022). Growth-defense trade-offs in plants. Curr. Biol..

[bib73] Hewezi T., Howe P., Maier T.R., Hussey R.S., Mitchum M.G., Davis E.L., Baum T.J. (2008). Cellulose binding protein from the parasitic nematode Heterodera schachtii interacts with Arabidopsis pectin methylesterase: cooperative cell wall modification during parasitism. Plant Cell.

[bib74] Hocher V., Alloisio N., Auguy F., Fournier P., Doumas P., Pujic P., Gherbi H., Queiroux C., Da Silva C., Wincker P. (2011). Transcriptomics of Actinorhizal Symbioses Reveals Homologs of the Whole Common Symbiotic Signaling Cascade. Plant Physiology.

[bib75] Hocq L., Habrylo O., Sénéchal F., Voxeur A., Pau-Roblot C., Safran J., Fournet F., Bassard S., Battu V., Demailly H. (2024). Mutation of AtPME2, a pH-Dependent Pectin Methylesterase, Affects Cell Wall Structure and Hypocotyl Elongation. Plant Cell Physiol..

[bib76] Hothorn M., D’Angelo I., Márquez J.A., Greiner S., Scheffzek K. (2004). The Invertase Inhibitor Nt-CIF from Tobacco: A Highly Thermostable Four-helix Bundle with an Unusual N-terminal Extension. J. Mol. Biol..

[bib77] Hothorn M., Wolf S., Aloy P., Greiner S., Scheffzek K. (2004). Structural insights into the target specificity of plant invertase and pectin methylesterase inhibitory proteins. Plant Cell.

[bib78] Hua L., Yong C., Zhanquan Z., Boqiang L., Guozheng Q., Shiping T. (2018). Pathogenic mechanisms and control strategies of Botrytis cinerea causing post-harvest decay in fruits and vegetables. Food Qual. Saf..

[bib79] Huang Y.-C., Niu C.-Y., Yang C.-R., Jinn T.-L. (2016). The Heat Stress Factor HSFA6b Connects ABA Signaling and ABA-Mediated Heat Responses. Plant Physiol.

[bib80] Huang Y.-C., Wu H.-C., Wang Y.-D., Liu C.-H., Lin C.-C., Luo D.-L., Jinn T.-L. (2017). PECTIN METHYLESTERASE34 Contributes to Heat Tolerance through Its Role in Promoting Stomatal Movement. Plant Physiol..

[bib81] Huang D., Mao Y., Guo G., Ni D., Chen L. (2022). Genome-wide identification of PME gene family and expression of candidate genes associated with aluminum tolerance in tea plant (Camellia sinensis). BMC Plant Biol..

[bib82] Huang D., Ma J., Chen X., Wang H., Tan R., Jiao L., Chen J., Mao Y., Chen L. (2025). CsWRKY17 enhances Al accumulation by promoting pectin deesterification in tea plant. Hortic. Res..

[bib83] Huerta A.I., Sancho-Andrés G., Montesinos J.C., Silva-Navas J., Bassard S., Pau-Roblot C., Kesten C., Schlechter R., Dora S., Ayupov T. (2023). The WAK-like protein RFO1 acts as a sensor of the pectin methylation status in Arabidopsis cell walls to modulate root growth and defense. Mol. Plant.

[bib84] Ibar C., Orellana A. (2007). The Import of S-Adenosylmethionine into the Golgi Apparatus Is Required for the Methylation of Homogalacturonan. Plant Physiology.

[bib85] Jia M., Yang H., Li B., Xie Z., Cao S., Pan L., Zhang B., Zhou Y. (2026). Root development progression involves RALF peptide-mediated ROS signaling and pectin dynamics in rice. Nat. Commun..

[bib86] Jiang Z., van Zanten M., Sasidharan R. (2025). Mechanisms of plant acclimation to multiple abiotic stresses. Commun. Biol..

[bib87] Jithesh M.N., Wally O.S.D., Manfield I., Critchley A.T., Hiltz D., Prithiviraj B. (2012). Analysis of Seaweed Extract-induced Transcriptome Leads to Identification of a Negative Regulator of Salt Tolerance in Arabidopsis. Hortscience.

[bib88] Johansson K., El-Ahmad M., Friemann R., Jörnvall H., Markovič O., Eklund H. (2002). Crystal structure of plant pectin methylesterase. FEBS Lett..

[bib89] Kan Y., Mu X.-R., Gao J., Lin H.-X., Lin Y. (2023). The molecular basis of heat stress responses in plants. Mol. Plant.

[bib90] Kim Y., Cameron R.G., Williams M.A.K., Luzio G.A. (2018). Structural and functional effects of manipulating the degree of methylesterification in a model homogalacturonan with a pseudo-random fungal pectin methylesterase followed by a processive methylesterase. Food Hydrocoll..

[bib91] Kim C.-Y., Song H., Lee Y.-H. (2022). Ambivalent response in pathogen defense: A double-edged sword?. Plant Commun..

[bib92] Kohn R., Markovič O., Machová E. (1983). Deesterification mode of pectin by pectin esterases of *Aspergillus foetidus*, tomatoes and alfalfa. Collect. Czech Chem. Commun..

[bib93] Kohorn B.D., Kohorn S.L., Saba N.J., Martinez V.M. (2014). Requirement for Pectin Methyl Esterase and Preference for Fragmented over Native Pectins for Wall-associated Kinase-activated, EDS1/PAD4-dependent Stress Response in Arabidopsis. J. Biol. Chem..

[bib94] Komarova T.V., Sheshukova E.V., Dorokhov Y.L. (2014). Cell wall methanol as a signal in plant immunity. Front. Plant Sci..

[bib95] Kong F., Yang L. (2023). Pathogen-triggered changes in plant development: Virulence strategies or host defense mechanism?. Front. Microbiol..

[bib96] Körner E., von Dahl C.C., Bonaventure G., Baldwin I.T. (2009). Pectin methylesterase NaPME1 contributes to the emission of methanol during insect herbivory and to the elicitation of defence responses in Nicotiana attenuata. J. Exp. Bot..

[bib97] Kračun S.K., Fangel J.U., Rydahl M.G., Pedersen H.L., Vidal-Melgosa S., Willats W.G.T. (2017). Carbohydrate Microarray Technology Applied to High-Throughput Mapping of Plant Cell Wall Glycans Using Comprehensive Microarray Polymer Profiling (CoMPP). Methods Mol. Biol..

[bib98] Lagrèze J., Pajuelo A.S., Coculo D., Rojas B., Pizzio G.A., Zhang C., Tian M.-B., Malnoy M., Vannozzi A., Costa L.D. (2025). PME10 Is a Pectin Methylesterase Driving PME Activity and Immunity Against Botrytis cinerea in Grapevine (Vitis vinifera L.). Plant Biotechnol. J..

[bib99] Lakshmanan V., Castaneda R., Rudrappa T., Bais H.P. (2013). Root transcriptome analysis of Arabidopsis thaliana exposed to beneficial Bacillus subtilis FB17 rhizobacteria revealed genes for bacterial recruitment and plant defense independent of malate efflux. Planta.

[bib236] Lazarowitz S.G., Beachy R.N. (1999). Viral movement proteins as probes for intracellular and intercellular trafficking in plants. Plant Cell.

[bib101] Levigne S., Thomas M., Ralet M.-C., Quemener B., Thibault J.-F. (2002). Determination of the degrees of methylation and acetylation of pectins using a C18 column and internal standards. Food Hydrocoll..

[bib102] Li Z., Wu L., Wang C., Wang Y., He L., Wang Z., Ma X., Bai F., Feng G., Liu J. (2022). Characterization of pectin methylesterase gene family and its possible role in juice sac granulation in navel orange (Citrus sinensis Osbeck). BMC Genom..

[bib103] Li A., Wang Y., Zou J., Yin J., Zhang S., Li X., Shen H., Liu J., Sun Z. (2024). Phosphorus deficiency-induced cell wall pectin demethylesterification enhances cadmium accumulation in roots of Salix caprea. J. Environ. Manag..

[bib104] Limberg G., Körner R., Buchholt H.C., Christensen T.M.I.E., Roepstorff P., Mikkelsen J.D. (2000). Quantification of the amount of galacturonic acid residues in blocksequences in pectin homogalacturonan by enzymatic fingerprinting with exo- and endo-polygalacturonase II from Aspergillus niger. Carbohydr. Res..

[bib105] Limberg G., Körner R., Buchholt H.C., Christensen T.M.I.E., Roepstorff P., Mikkelsen J.D. (2000). Analysis of different de-esterification mechanisms for pectin by enzymatic fingerprinting using endopectin lyase and endopolygalacturonase II from A. niger. Carbohydr. Res..

[bib106] Lin H., Ye H., Shao Q., Cheng S., Bi G., Lin B., Lin H., He L., Shen B., Zhu H. (2025). Genome-Wide Analyses and Expression Profiling of PME/PMEI Gene Families Reveal Their Relevance to Chilling Stress Response and Grafted Healing Efficiency in Cucumber/Pumpkin-Grafted Plants. Plants.

[bib107] Lionetti V. (2015). PECTOPLATE: the simultaneous phenotyping of pectin methylesterases, pectinases, and oligogalacturonides in plants during biotic stresses. Front. Plant Sci..

[bib108] Lionetti V., Raiola A., Camardella L., Giovane A., Obel N., Pauly M., Favaron F., Cervone F., Bellincampi D. (2007). Overexpression of Pectin Methylesterase Inhibitors in Arabidopsis Restricts Fungal Infection by Botrytis cinerea. Plant Physiol..

[bib109] Lionetti V., Francocci F., Ferrari S., Volpi C., Bellincampi D., Galletti R., D’Ovidio R., De Lorenzo G., Cervone F. (2010). Engineering the cell wall by reducing de-methyl-esterified homogalacturonan improves saccharification of plant tissues for bioconversion. Proc. Natl. Acad. Sci. USA.

[bib110] Lionetti V., Cervone F., Bellincampi D. (2012). Methyl esterification of pectin plays a role during plant-pathogen interactions and affects plant resistance to diseases. J. Plant Physiol..

[bib111] Lionetti V., Raiola A., Cervone F., Bellincampi D. (2014). How do pectin methylesterases and their inhibitors affect the spreading of tobamovirus?. Plant Signal. Behav..

[bib112] Lionetti V., Raiola A., Cervone F., Bellincampi D. (2014). Transgenic expression of pectin methylesterase inhibitors limits tobamovirus spread in tobacco and Arabidopsis: PMEIs limit tobamovirus infection. Mol. Plant Pathol..

[bib113] Lionetti V., Giancaspro A., Fabri E., Giove S.L., Reem N., Zabotina O.A., Blanco A., Gadaleta A., Bellincampi D. (2015). Cell wall traits as potential resources to improve resistance of durum wheat against Fusarium graminearum. BMC Plant Biol..

[bib114] Lionetti V., Fabri E., De Caroli M., Hansen A.R., Willats W.G., Piro G., Bellincampi D. (2017). Three pectin methyl esterase inhibitors protect cell wall integrity for immunity to Botrytis. Plant Physiol.

[bib115] Liu N., Sun Y., Pei Y., Zhang X., Wang P., Li X., Li F., Hou Y. (2018). A Pectin Methylesterase Inhibitor Enhances Resistance to Verticillium Wilt1[OPEN]. Plant Physiol..

[bib116] Liu H., Liu L., Liang D., Zhang M., Jia C., Qi M., Liu Y., Shao Z., Meng F., Hu S. (2021). SlBES1 promotes tomato fruit softening through transcriptional inhibition of *PMEU1*. iScience.

[bib117] Liu M.-C.J., Yeh F.-L.J., Yvon R., Simpson K., Jordan S., Chambers J., Wu H.-M., Cheung A.Y. (2024). Extracellular pectin-RALF phase separation mediates FERONIA global signaling function. Cell.

[bib118] Manrique G.D., Lajolo F.M. (2002). FT-IR spectroscopy as a tool for measuring degree of methyl esterification in pectins isolated from ripening papaya fruit. Postharvest Biol. Technol..

[bib119] Manteau S., Abouna S., Lambert B., Legendre L. (2003). Differential regulation by ambient pH of putative virulence factor secretion by the phytopathogenic fungus Botrytis cinerea. FEMS Microbiol. Ecol..

[bib120] Mareck A., Lamour R., Schaumann A., Chan P., Driouich A., Pelloux J., Lerouge P. (2012). Analysis of LuPME3, a pectin methylesterase from Linum usitatissimum, revealed a variability in PME proteolytic maturation. Plant Signal. Behav..

[bib121] Markovič O., Janeček Š. (2004). Pectin methylesterases: sequence-structural features and phylogenetic relationships. Carbohydr. Res..

[bib122] Marty P., Jouan B., Bertheau Y., Vian B., Goldberg R. (1997). Charge density in stem cell walls of Solanum tuberosum genotypes and susceptibility to blackleg. Phytochemistry.

[bib123] McMillan G.P., Hedley D., Fyffe L., Pérombelon M.C.M. (1993). Potato resistance to soft-rot erwinias is related to cell wall pectin esterification. Physiol. Mol. Plant Pathol..

[bib124] Men S., Zhao F., Zhang X., Guan Z., Xu J., Xue Y., Tan H., Chang X., Zhao G., Li C. (2026). Expansin-like A binds to pectin via electrostatic forces and remodels the plant cell wall. Plant Physiol..

[bib125] Mercadante D., Melton L.D., Jameson G.B., Williams M.A.K. (2014). Processive Pectin Methylesterases: The Role of Electrostatic Potential, Breathing Motions and Bond Cleavage in the Rectification of Brownian Motions. PLoS One.

[bib126] Meyer M., Huttenlocher F., Cedzich A., Procopio S., Stroeder J., Pau-Roblot C., Lequart-Pillon M., Pelloux J., Stintzi A., Schaller A. (2016). The subtilisin-like protease SBT3 contributes to insect resistance in tomato. J. Exp. Bot..

[bib127] Micheli F. (2001). Pectin methylesterases: cell wall enzymes with important roles in plant physiology. Trends Plant Sci..

[bib128] Mohnen D. (2008). Pectin structure and biosynthesis. Curr. Opin. Plant Biol..

[bib129] Molina A., Jordá L., Torres M.Á., Martín-Dacal M., Berlanga D.J., Fernández-Calvo P., Gómez-Rubio E., Martín-Santamaría S. (2024). Plant cell wall-mediated disease resistance: Current understanding and future perspectives. Mol. Plant.

[bib130] Moller I., Marcus S.E., Haeger A., Verhertbruggen Y., Verhoef R., Schols H., Ulvskov P., Mikkelsen J.D., Knox J.P., Willats W. (2008). High-throughput screening of monoclonal antibodies against plant cell wall glycans by hierarchical clustering of their carbohydrate microarray binding profiles. Glycoconj. J..

[bib131] Mouille G., Ralet M., Cavelier C., Eland C., Effroy D., Hématy K., McCartney L., Truong H.N., Gaudon V., Thibault J. (2007). Homogalacturonan synthesis in *Arabidopsis thaliana* requires a Golgi-localized protein with a putative methyltransferase domain. Plant J..

[bib132] Moussu S., Lee H.K., Haas K.T., Broyart C., Rathgeb U., De Bellis D., Levasseur T., Schoenaers S., Fernandez G.S., Grossniklaus U. (2023). Plant cell wall patterning and expansion mediated by protein-peptide-polysaccharide interaction. Science.

[bib133] Munzert K.S., Engelsdorf T. (2025). Plant cell wall structure and dynamics in plant–pathogen interactions and pathogen defence. J. Exp. Bot..

[bib134] Nafisi M., Fimognari L., Sakuragi Y. (2015). Interplays between the cell wall and phytohormones in interaction between plants and necrotrophic pathogens. Phytochemistry.

[bib135] Nemecekmarshall M., Macdonald R.C., Franzen J.J., Wojciechowski C.L., Fall R. (1995). Methanol Emission from Leaves - Enzymatic Detection of Gas-Phase Methanol and Relation of Methanol Fluxes to Stomatal Conductance and Leaf Development. Plant Physiol..

[bib136] Neubauer J.D., Lulai E.C., Thompson A.L., Suttle J.C., Bolton M.D. (2012). Wounding coordinately induces cell wall protein, cell cycle and pectin methyl esterase genes involved in tuber closing layer and wound periderm development. J. Plant Physiol..

[bib137] Obomighie I., Prentice I.J., Lewin-Jones P., Bachtiger F., Ramsay N., Kishi-Itakura C., Goldberg M.W., Hawkins T.J., Sprittles J.E., Knight H., Sosso G.C. (2025). Understanding pectin cross-linking in plant cell walls. Commun. Biol..

[bib138] O’Neill M.A., Black I., Urbanowicz B., Bharadwaj V., Crowley M., Koj S., Peña M.J. (2020). Locating Methyl-Etherified and Methyl-Esterified Uronic Acids in the Plant Cell Wall Pectic Polysaccharide Rhamnogalacturonan II. SLAS Technology.

[bib139] Osakabe Y., Osakabe K., Shinozaki K., Tran L.-S.P. (2014). Response of plants to water stress. Front. Plant Sci..

[bib140] Osorio S., Castillejo C., Quesada M.A., Medina-Escobar N., Brownsey G.J., Suau R., Heredia A., Botella M.A., Valpuesta V. (2008). Partial demethylation of oligogalacturonides by pectin methyl esterase 1 is required for eliciting defence responses in wild strawberry (Fragaria vesca). Plant J..

[bib141] Osorio S., Bombarely A., Giavalisco P., Usadel B., Stephens C., Aragüez I., Medina-Escobar N., Botella M.A., Fernie A.R., Valpuesta V. (2011). Demethylation of oligogalacturonides by FaPE1 in the fruits of the wild strawberry Fragaria vesca triggers metabolic and transcriptional changes associated with defence and development of the fruit. J. Exp. Bot..

[bib143] Pattathil S., Avci U., Baldwin D., Swennes A.G., McGill J.A., Popper Z., Bootten T., Albert A., Davis R.H., Chennareddy C. (2010). A Comprehensive Toolkit of Plant Cell Wall Glycan-Directed Monoclonal Antibodies. Plant Physiology.

[bib144] Pattathil S., Avci U., Miller J.S., Hahn M.G. (2012). Immunological approaches to plant cell wall and biomass characterization: Glycome Profiling. Methods Mol. Biol..

[bib145] Paynel F., Schaumann A., Arkoun M., Douchiche O., Morvan C. (2009). Temporal regulation of cell-wall pectin methylesterase and peroxidase isoforms in cadmium-treated flax hypocotyl. Ann. Bot..

[bib146] Peaucelle A., Louvet R., Johansen J.N., Salsac F., Morin H., Fournet F., Belcram K., Gillet F., Höfte H., Laufs P. (2011). The transcription factor BELLRINGER modulates phyllotaxis by regulating the expression of a pectin methylesterase in Arabidopsis. Development.

[bib147] Peña M.J., Ryden P., Madson M., Smith A.C., Carpita N.C. (2004). The galactose residues of xyloglucan are essential to maintain mechanical strength of the primary cell walls in Arabidopsis during growth. Plant Physiol..

[bib148] Peñuelas J., Filella I., Stefanescu C., Llusià J. (2005). Caterpillars of Euphydryas aurinia (Lepidoptera: Nymphalidae) feeding on Succisa pratensis leaves induce large foliar emissions of methanol. New Phytol..

[bib149] Pilling J., Willmitzer L., Fisahn J. (2000). Expression of a Petunia inflata pectin methyl esterase in Solanum tuberosum L. enhances stem elongation and modifies cation distribution. Planta.

[bib150] Pilling J., Willmitzer L., Bucking H., Fisahn J. (2004). Inhibition of a ubiquitously expressed pectin methyl esterase in Solanum tuberosum L. affects plant growth, leaf growth polarity, and ion partitioning. Planta.

[bib151] Plattner S., Gruber C., Altmann F., Bohlmann H. (2014). Self-processing of a barley subtilase expressed in E. coli. Protein Expr. Purif..

[bib152] Plazinski W. (2011). Molecular basis of calcium binding by polyguluronate chains. Revising the egg-box model. J. Comput. Chem..

[bib153] Pogorelko G., Lionetti V., Bellincampi D., Zabotina O. (2013). Cell wall integrity: Targeted post-synthetic modifications to reveal its role in plant growth and defense against pathogens. Plant Signal. Behav..

[bib154] Pogorelko G.V., Juvale P.S., Rutter W.B., Hütten M., Maier T.R., Hewezi T., Paulus J., van der Hoorn R.A., Grundler F.M., Siddique S. (2019). Re-targeting of a plant defense protease by a cyst nematode effector. Plant J..

[bib155] Prusky D., Yakoby N. (2003). Pathogenic fungi: leading or led by ambient pH?. Mol. Plant Pathol..

[bib156] Qi W., Ye T., Xiaolong Z., Xiaoying D., Jixing X., Renfang S., Xiaofang Z. (2022). Pectin Methylesterases Enhance Root Cell Wall Phosphorus Remobilization in Rice. Rice Sci..

[bib157] Qu T., Liu R., Wang W., An L., Chen T., Liu G., Zhao Z. (2011). Brassinosteroids regulate pectin methylesterase activity and AtPME41 expression in Arabidopsis under chilling stress. Cryobiology.

[bib158] Raiola A., Lionetti V., Elmaghraby I., Immerzeel P., Mellerowicz E.J., Salvi G., Cervone F., Bellincampi D. (2011). Pectin methylesterase is induced in Arabidopsis upon infection and is necessary for a successful colonization by necrotrophic pathogens. Mol. Plant Microbe Interact..

[bib159] Ramírez V., López A., Mauch-Mani B., Gil M.J., Vera P. (2013). An Extracellular Subtilase Switch for Immune Priming in Arabidopsis. PLoS Pathog..

[bib160] Rashid A. (2016). Defense responses of plant cell wall non-catalytic proteins against pathogens. Physiol. Mol. Plant Pathol..

[bib161] Reem N.T., Pogorelko G., Lionetti V., Chambers L., Held M.A., Bellincampi D., Zabotina O.A. (2016). Decreased polysaccharide feruloylation compromises plant cell wall integrity and increases susceptibility to necrotrophic fungal pathogens. Front. Plant Sci..

[bib162] Ren C., Kermode A.R. (2000). An Increase in Pectin Methyl Esterase Activity Accompanies Dormancy Breakage and Germination of Yellow Cedar Seeds. Plant Physiol..

[bib163] Rondeau-Mouro C., Defer D., Leboeuf E., Lahaye M. (2008). Assessment of cell wall porosity in *Arabidopsis thaliana* by NMR spectroscopy. Int. J. Biol. Macromol..

[bib164] Rößling A.-K., Dünser K., Liu C., Lauw S., Rodriguez-Franco M., Kalmbach L., Barbez E., Kleine-Vehn J. (2024). Pectin methylesterase activity is required for RALF1 peptide signalling output. eLife.

[bib165] Rui Y., Dinneny J.R. (2020). A wall with integrity: surveillance and maintenance of the plant cell wall under stress. New Phytol..

[bib166] Rzemieniewski J., Stegmann M. (2022). Regulation of pattern-triggered immunity and growth by phytocytokines. Curr. Opin. Plant Biol..

[bib167] Savary B.J., Nuñez A. (2003). Gas chromatography–mass spectrometry method for determining the methanol and acetic acid contents of pectin using headspace solid-phase microextraction and stable isotope dilution. J. Chromatogr. A.

[bib168] Schoenaers S., Balcerowicz D., Breen G., Hill K., Zdanio M., Mouille G., Holman T.J., Oh J., Wilson M.H., Nikonorova N. (2018). The Auxin-Regulated CrRLK1L Kinase ERULUS Controls Cell Wall Composition during Root Hair Tip Growth. Curr. Biol..

[bib169] Schoenaers S., Lee H.K., Gonneau M., Faucher E., Levasseur T., Akary E., Claeijs N., Moussu S., Broyart C., Balcerowicz D. (2024). Rapid alkalinization factor 22 has a structural and signalling role in root hair cell wall assembly. Nat. Plants.

[bib170] Seifert G.J., Blaukopf C. (2010). Irritable Walls: The Plant Extracellular Matrix and Signaling1. Plant Physiol..

[bib171] Sénéchal F., Graff L., Surcouf O., Marcelo P., Rayon C., Bouton S., Mareck A., Mouille G., Stintzi A., Höfte H. (2014). Arabidopsis PECTIN METHYLESTERASE17 is co-expressed with and processed by SBT3.5, a subtilisin-like serine protease. Ann. Bot..

[bib172] Sénéchal F., Habrylo O., Hocq L., Domon J.-M., Marcelo P., Lefebvre V., Pelloux J., Mercadante D. (2017). Structural and dynamical characterization of the pH-dependence of the pectin methylesterase–pectin methylesterase inhibitor complex. J. Biol. Chem..

[bib173] Shikata H., Yoshinari A., Asaoka M., Takahashi D. (2026). Memory in the wall: expanding our understanding of the roles of plant cell walls. New Phytol..

[bib174] Silva-Sanzana C., Celiz-Balboa J., Garzo E., Marcus S.E., Parra-Rojas J.P., Rojas B., Olmedo P., Rubilar M.A., Rios I., Chorbadjian R.A. (2019). Pectin Methylesterases Modulate Plant Homogalacturonan Status in Defenses against the Aphid Myzus persicae. Plant Cell.

[bib175] Solecka D., Żebrowski J., Kacperska A. (2008). Are Pectins Involved in Cold Acclimation and De-acclimation of Winter Oil-seed Rape Plants?. Ann. Bot..

[bib176] Stavolone L., Lionetti V. (2017). Extracellular Matrix in Plants and Animals: Hooks and Locks for Viruses. Front. Microbiol..

[bib177] Stefanowicz K., Szymanska-Chargot M., Truman W., Walerowski P., Olszak M., Augustyniak A., Kosmala A., Zdunek A., Malinowski R. (2021). Plasmodiophora brassicae-Triggered Cell Enlargement and Loss of Cellular Integrity in Root Systems Are Mediated by Pectin Demethylation. Front. Plant Sci..

[bib178] Stegmann M., Monaghan J., Smakowska-Luzan E., Rovenich H., Lehner A., Holton N., Belkhadir Y., Zipfel C. (2017). The receptor kinase FER is a RALF-regulated scaffold controlling plant immune signaling. Science.

[bib179] Su C., Zhang G., Rodriguez-Franco M., Hinnenberg R., Wietschorke J., Liang P., Yang W., Uhler L., Li X., Ott T. (2023). Transcellular progression of infection threads in Medicago truncatula roots is associated with locally confined cell wall modifications. Curr. Biol..

[bib180] Sun J., Tian Z., Li X., Li S., Li Z., Wang J., Hu Z., Chen H., Guo C., Xie M., Xu R. (2022). Systematic analysis of the pectin methylesterase gene family in Nicotiana tabacum and reveal their multiple roles in plant development and abiotic stresses. Front. Plant Sci..

[bib181] Sun D., Lei Z., Carriquí M., Zhang Y., Liu T., Wang S., Song K., Zhu L., Zhang W., Zhang Y. (2025). Reductions in mesophyll conductance under drought stress are influenced by increases in cell wall chelator-soluble pectin content and denser microfibril alignment in cotton. J. Exp. Bot..

[bib182] Swaminathan S., Reem N.T., Lionetti V., Zabotina O.A. (2021). Coexpression of Fungal Cell Wall-Modifying Enzymes Reveals Their Additive Impact on Arabidopsis Resistance to the Fungal Pathogen, Botrytis cinerea. Biology.

[bib183] Swaminathan S., Lionetti V., Zabotina O.A. (2022). Plant Cell Wall Integrity Perturbations and Priming for Defense. Plants.

[bib184] Takahashi D., Willick I.R., Kasuga J., Livingston III D.P. (2021). Responses of the Plant Cell Wall to Sub-Zero Temperatures: A Brief Update. Plant Cell Physiol..

[bib185] Taurino M., Abelenda J.A., Río-Alvarez I., Navarro C., Vicedo B., Farmaki T., Jiménez P., García-Agustín P., López-Solanilla E., Prat S. (2014). Jasmonate-dependent modifications of the pectin matrix during potato development function as a defense mechanism targeted by Dickeya dadantii virulence factors. Plant J..

[bib186] Temple H., Phyo P., Yang W., Lyczakowski J.J., Echevarría-Poza A., Yakunin I., Parra-Rojas J.P., Terrett O.M., Saez-Aguayo S., Dupree R. (2022). Golgi-localized putative S-adenosyl methionine transporters required for plant cell wall polysaccharide methylation. Nat. Plants.

[bib187] Tibbits C.W., MacDougall A.J., Ring S.G. (1998). Calcium binding and swelling behaviour of a high methoxyl pectin gel. Carbohydr. Res..

[bib188] Tran D., Dauphin A., Meimoun P., Kadono T., Nguyen H.T.H., Arbelet-Bonnin D., Zhao T., Errakhi R., Lehner A., Kawano T., Bouteau F. (2018). Methanol induces cytosolic calcium variations, membrane depolarization and ethylene production in arabidopsis and tobacco. Ann. Bot..

[bib189] Traubenik S., Charon C., Blein T. (2024). From environmental responses to adaptation: the roles of plant lncRNAs. Plant Physiology.

[bib190] Tundo S., Kalunke R., Janni M., Volpi C., Lionetti V., Bellincampi D., Favaron F., D’Ovidio R. (2016). Pyramiding PvPGIP2 and TAXI-III But Not PvPGIP2 and PMEI Enhances Resistance Against Fusarium graminearum. Mol. Plant Microbe Interact..

[bib191] Ullah A., Lin Y.-J., Zhang H., Yu X.-Z. (2023). Identification of the Key Genes Involved in Proline-Mediated Modification of Cell Wall Components in Rice Seedlings under Trivalent Chromium Exposure. Toxics.

[bib192] Vicré M., Lionetti V. (2023). Editorial: Plant cell wall in pathogenesis, parasitism and symbiosis, Volume II. Front. Plant Sci..

[bib193] Vogel J.P., Raab T.K., Schiff C., Somerville S.C. (2002). PMR6, a pectate lyase-like gene required for powdery mildew susceptibility in Arabidopsis. Plant Cell.

[bib194] Volpi C., Janni M., Lionetti V., Bellincampi D., Favaron F., D’Ovidio R. (2011). The Ectopic Expression of a Pectin Methyl Esterase Inhibitor Increases Pectin Methyl Esterification and Limits Fungal Diseases in Wheat. Mol. Plant Microbe Interact..

[bib195] Von Dahl C.C., Hävecker M., Schlögl R., Baldwin I.T. (2006). Caterpillar-elicited methanol emission: a new signal in plant–herbivore interactions?. Plant J..

[bib196] Voxeur A., Habrylo O., Guénin S., Miart F., Soulié M.-C., Rihouey C., Pau-Roblot C., Domon J.-M., Gutierrez L., Pelloux J. (2019). Oligogalacturonide production upon Arabidopsis thaliana–Botrytis cinerea interaction. Proc. Natl. Acad. Sci..

[bib197] Wang X., Chung K.P., Lin W., Jiang L. (2017). Protein secretion in plants: conventional and unconventional pathways and new techniques. J. Exp. Bot..

[bib198] Wang S., Li R., Zhou Y., Fernie A.R., Ding Z., Zhou Q., Che Y., Yao Y., Liu J., Wang Y. (2023). Integrated Characterization of Cassava (Manihot esculenta) Pectin Methylesterase (MePME) Genes to Filter Candidate Gene Responses to Multiple Abiotic Stresses. Plants.

[bib199] Wang Y., Peng Y., Shangguan X., Yan J., Yu X., Jing W., Peng K., Chen Y., Shen Z., Xia Y. (2025). The pectin methylesterase OsPME14 modifies the cell wall to confer copper tolerance in *Oryza sativa* L. Plant J..

[bib200] Wang D., Ortega-Salazar I.B., Blanco-Ulate B. (2025). Homogalacturonan Methylesterification and Cell Wall Regulation: Integrating Biochemistry, Mechanics, and Developmental Signaling for Crop Improvement. Agronomy.

[bib201] Weber M., Deinlein U., Fischer S., Rogowski M., Geimer S., Tenhaken R., Clemens S. (2013). A mutation in the Arabidopsis thaliana cell wall biosynthesis gene pectin methylesterase 3 as well as its aberrant expression cause hypersensitivity specifically to Zn. Plant J..

[bib202] Weng X., Wang H., Jiang Y. (2025). NtLLG4-mediated unconventional polar exocytosis of NtPPME1 coordinates cell wall rigidity and membrane dynamics to control pollen tube integrity. Sci. Adv..

[bib203] Willats W.G.T., Orfila C., Limberg G., Buchholt H.C., van Alebeek G.J.W.M., Voragen A.G., Marcus S.E., Christensen T.M.I.E., Mikkelsen J.D., Murray B.S., Knox J.P. (2001). Modulation of the degree and pattern of methyl esterification of pectic homogalacturonan in plant cell walls: implications for pectin methyl esterase action, matrix properties and cell adhesion. J. Biol. Chem..

[bib204] Wolf S., Rausch T., Greiner S. (2009). The N-terminal pro region mediates retention of unprocessed type-I PME in the Golgi apparatus. Plant J..

[bib205] Wolf S., van der Does D., Ladwig F., Sticht C., Kolbeck A., Schürholz A.-K., Augustin S., Keinath N., Rausch T., Greiner S. (2014). A receptor-like protein mediates the response to pectin modification by activating brassinosteroid signaling. Proc. Natl. Acad. Sci. USA.

[bib206] Wu H.-C., Jinn T.-L. (2010). Heat shock-triggered Ca2+ mobilization accompanied by pectin methylesterase activity and cytosolic Ca2+ oscillation are crucial for plant thermotolerance. Plant Signal. Behav..

[bib207] Wu H.-C., Huang Y.-C., Stracovsky L., Jinn T.-L. (2017). Pectin methylesterase is required for guard cell function in response to heat. Plant Signal. Behav..

[bib208] Wu H.-C., Bulgakov V.P., Jinn T.-L. (2018). Pectin Methylesterases: Cell Wall Remodeling Proteins Are Required for Plant Response to Heat Stress. Front. Plant Sci..

[bib209] Wu H.-C., Yu S.-Y., Wang Y.-D., Jinn T.-L. (2022). Guard Cell-Specific Pectin METHYLESTERASE53 Is Required for Abscisic Acid-Mediated Stomatal Function and Heat Response in Arabidopsis. Front. Plant Sci..

[bib210] Wu H.-C., Yu S.-Y., Vivek S., Wang Y.-D., Jinn T.-L. (2025). ABA-mediated regulation of PME12 influences stomatal density, pore aperture, and heat stress response in Arabidopsis thaliana. Planta.

[bib211] Wydra K., Beri H. (2006). Structural changes of homogalacturonan, rhamnogalacturonan I and arabinogalactan protein in xylem cell walls of tomato genotypes in reaction to Ralstonia solanacearum. Physiol. Mol. Plant Pathol..

[bib212] Xia Y., Sun G., Xiao J., He X., Jiang H., Zhang Z., Zhang Q., Li K., Zhang S., Shi X. (2024). AlphaFold-guided redesign of a plant pectin methylesterase inhibitor for broad-spectrum disease resistance. Mol. Plant.

[bib213] Xiao Y., Sun G., Yu Q., Gao T., Zhu Q., Wang R., Huang S., Han Z., Cervone F., Yin H. (2024). A plant mechanism of hijacking pathogen virulence factors to trigger innate immunity. Science.

[bib214] Xu Y., Wang Y., Wang X., Pei S., Kong Y., Hu R., Zhou G. (2020). Transcription Factors BLH2 and BLH4 Regulate Demethylesterification of Homogalacturonan in Seed Mucilage. Plant Physiol..

[bib215] Xu Z., Gao Y., Gao C., Mei J., Wang S., Ma J., Yang H., Cao S., Wang Y., Zhang F. (2022). Glycosylphosphatidylinositol anchor lipid remodeling directs proteins to the plasma membrane and governs cell wall mechanics. Plant Cell.

[bib216] Yan J., He H., Fang L., Zhang A. (2018). Pectin methylesterase31 positively regulates salt stress tolerance in Arabidopsis. Biochem. Biophys. Res. Commun..

[bib217] Yang X.Y., Zeng Z.H., Yan J.Y., Fan W., Bian H.W., Zhu M.Y., Yang J.L., Zheng S.J. (2013). Association of specific pectin methylesterases with Al-induced root elongation inhibition in rice. Physiol. Plantarum.

[bib218] Yang Y., Yu Y., Liang Y., Anderson C.T., Cao J. (2018). A Profusion of Molecular Scissors for Pectins: Classification, Expression, and Functions of Plant Polygalacturonases. Front. Plant Sci..

[bib219] Yang W., Ruan M., Xiang M., Deng A., Du J., Xiao C. (2020). Overexpression of a pectin methylesterase gene PtoPME35 from Populus tomentosa influences stomatal function and drought tolerance in Arabidopsis thaliana. Biochem. Biophys. Res. Commun..

[bib220] Yang H., Kim X., Skłenar J., Aubourg S., Sancho-Andrés G., Stahl E., Guillou M.-C., Gigli-Bisceglia N., Tran Van Canh L., Bender K.W. (2023). Subtilase-mediated biogenesis of the expanded family of SERINE RICH ENDOGENOUS PEPTIDES. Nat. Plants.

[bib221] Yang Z., Wang M., Fan S., Zhang Z., Zhang D., He J., Li T., Wei R., Wang P., Dawood M. (2024). GhPME36 aggravates susceptibility to Liriomyza sativae by affecting cell wall biosynthesis in cotton leaves. BMC Biol..

[bib222] Yu X., Yang Z., Xu Y., Wang Z., Fan C., Zeng X., Liu Y., Lei T., Jiang M., Li J. (2023). Effect of chromium stress on metal accumulation and cell wall fractions in Cosmos bipinnatus. Chemosphere.

[bib223] Zhang D., Du Y., He D., Zhou D., Wu J., Peng J., Liu L., Liu Z., Yan M. (2021). Use of Comparative Transcriptomics Combined With Physiological Analyses to Identify Key Factors Underlying Cadmium Accumulation in Brassica juncea L. Front. Genet..

[bib224] Zhang L., Liu J., Cheng J., Sun Q., Zhang Y., Liu J., Li H., Zhang Z., Wang P., Cai C. (2022). lncRNA7 and lncRNA2 modulate cell wall defense genes to regulate cotton resistance to Verticillium wilt. Plant Physiology.

[bib225] Zhang X., Guo H., Xiao C., Yan Z., Ning N., Chen G., Zhang J., Hu H. (2023). PECTIN METHYLESTERASE INHIBITOR18 functions in stomatal dynamics and stomatal dimension. Plant Physiol..

[bib226] Zhang C., Xie Y., He P., Shan L. (2024). Unlocking Nature’s Defense: Plant Pattern Recognition Receptors as Guardians Against Pathogenic Threats. MPMI (Mol. Plant-Microbe Interact.).

[bib227] Zhang S., Yuan X., Duan J., Hu J., Wei C., Zhang Y., Wang J., Li C., Hou S., Luo X. (2024). Genome-wide identification and characterization of pectin methylesterase inhibitor gene family members related to abiotic stresses in watermelon. Front. Plant Sci..

[bib228] Zhang T., Yu L., Wang Y., Li P., Feng X., Jian G., Zhao F., Liu X., Yang Z., Sha X. (2026). Esterified-pectin-coupled polar stiffening controls grass stomatal opening. Nat. Plants.

[bib229] Zhou K. (2022). The regulation of the cell wall by glycosylphosphatidylinositol-anchored proteins in Arabidopsis. Front. Cell Dev. Biol..

[bib230] Zhou T., Wu P.j., Chen J.f., Du X.q., Feng Y.n., Hua Y.p. (2024). Pectin demethylation-mediated cell wall Na+ retention positively regulates salt stress tolerance in oilseed rape. Theor. Appl. Genet..

[bib231] Zhu H., Wang J., Huang R., Yang Z., Fan W., Huang L., Yang J., Chen W. (2024). Epigenetic modification of a pectin methylesterase gene activates apoplastic iron reutilization in tomato roots. Plant Physiology.

[bib232] Zhu Y., Wang X., He Y., Liu Y., Wang R., Liu Y., Wang S. (2024). Chromosome doubling increases *PECTIN METHYLESTERASE 2* expression, biomass, and osmotic stress tolerance in kiwifruit. Plant Physiology.

